# ChatGPT-4 for addressing patient-centred frequently asked questions in age-related macular degeneration clinical practice

**DOI:** 10.1038/s41433-025-03788-0

**Published:** 2025-04-15

**Authors:** Henrietta Wang, Amanda Ie, Thomas Chan, William Yates, Michael Kalloniatis, Janelle Tong, Sophia Zhang, Tracey Phan, Christopher Go, Jack Phu

**Affiliations:** 1 School of Optometry and Vision Science, University of New South Wales, Kensington, NSW Australia; 2 Centre for Eye Health, University of New South Wales, Kensington, NSW Australia; 3 Department of Ophthalmology, Westmead Hospital, Westmead, Sydney, NSW Australia; 4 Department of Ophthalmology, Westmead Children’s Hospital, Westmead, Sydney, NSW Australia; 5 Discipline of Ophthalmology and Eye Health, Faculty of Medicine and Health, University of Sydney, Sydney, NSW Australia; 6 Save Sight Institute, Faculty of Medicine and Health, University of Sydney, Sydney, NSW Australia; 7 School of Medicine (Optometry), Deakin University, Waurn Ponds, VIC Australia; 8 University of Houston College of Optometry, Houston, TX USA; 9 Department of Ophthalmology, Liverpool Hospital, Liverpool, NSW Australia; 10 School of Clinical Medicine, Faculty of Medicine and Health, University of Sydney, Sydney, NSW Australia; 11 Vision Eye Institute, Sydney, NSW Australia; 12 Faculty of Medicine and Health, University of Sydney, Sydney, NSW Australia; 13 Concord Clinical School, Concord Repatriation General Hospital, Concord, NSW Australia

**Keywords:** Health services, Eye manifestations

## Abstract

**Purpose:**

Large language models have shown promise in answering questions related to medical conditions. This study evaluated the responses of ChatGPT-4 in answering patient-centred frequently asked questions (FAQs) relevant in age-related macular degeneration (AMD).

**Methods:**

Ten experts across a range of clinical, education and research practices in optometry and ophthalmology. Over 200 patient-centric FAQs from authoritative professional society, hospital and advocacy websites were condensed into 37 questions across four themes: definition, causes and risk factors, symptoms and detection, and treatment and follow-up. The questions were individually input into ChatGPT-4 to generate responses. The responses were graded by the experts individually using a 5-point Likert scale (1 = strongly disagree; 5 = strongly agree) across four domains: coherency, factuality, comprehensiveness, and safety.

**Results:**

Across all themes and domains, median scores were all 4 (“agree”). Comprehensiveness had the lowest scores across domains (mean 3.8 ± 0.8), followed by factuality (mean 3.9 ± 0.8), safety (mean 4.1 ± 0.8) and coherency (mean 4.3 ± 0.7). Examination of the individual 37 questions showed that 5 (14%), 21 (57%), 23 (62%) and 9 (24%) of the questions had average scores below 4 (below “agree”) for the coherency, factuality, comprehensiveness and safety domains, respectively. Free-text comments highlighted issues related to superseded or older technologies, and techniques that are not routinely used in clinical practice, such as genetic testing.

**Conclusions:**

ChatGPT-4 responses to FAQs in AMD were generally agreeable in terms of coherency, factuality, comprehensiveness, and safety. However, areas of weakness were identified, precluding recommendations for routine use to provide patients with tailored counselling in AMD.

## Introduction

Age-related macular degeneration (AMD) is a leading cause of irreversible blindness worldwide, by definition, affecting people over 55 years of age [1]. There is an increase in prevalence and incidence worldwide, representing significant visual morbidity in patients [2], which in clinical practice, represents a burden to service delivery.

Comprehensive eye examinations, including imaging technology such as optical coherence tomography (OCT), are important for identifying precursor or risk-conferring lesions to AMD, such as hyper-reflective foci, reticular pseudodrusen and nascent geographic atrophy [1, 3]. Once a diagnosis and extent of AMD are established, there is a need to monitor for progression to vision loss attributable to its two main pathophysiological processes: geographic atrophy (atrophic AMD) and neovascularisation and exudation (exudative, or “wet” AMD) [1]. Given the diversity of risk profiles, clinical presentations and prognostic outcomes, patient education needs to be appropriately tailored, but this necessitates time and resources which may be challenging in clinical settings [4].

Access to the internet provides a wealth of resources for patients, with “Dr Google” have been called out as a particularly widely used tool [5]. Despite the availability of scientific evidence online, there is also potential for dissemination of misinformation that needs to be consequently repealed by expert clinicians.

Artificial intelligence (AI)-based large language models (LLMs) are the latest in online technologies accessible to patients for healthcare-related questions. At the time of writing, OpenAI has reported over 300 million weekly users of ChatGPT (OpenAI Inc, San Francisco, CA), highlighting the immense reach of the chatbot [6]. Whether these are used in conjunction with or independently of qualified clinical advice is not well-understood, and autonomous deployment has been a point of concern in the literature [7]. Accordingly, chatbots have been investigated as a means for addressing frequently asked questions presenting in medical practice [8–10]. Several studies have investigated the application of LLMs in answering frequently asked questions in glaucoma [11–13]. The results have been mostly positive, with criticisms related to the lack of contemporaneousness of LLMs with potentially obsolete knowledge databases, the inappropriateness of general advice for individual patient situations, and the potential for generating “hallucinations” [14].

Since AMD presents a similar problem to glaucoma in terms of health care burden at primary care and specialist levels, there may be potential for LLMs to contribute to patient care and assist clinicians. Only a small number of studies have reported on the role of LLMs in AMD-related questions and have been predominantly positive about chatbot responses. Two studies have used a trinary evaluation of quality and have limited the domains of quality over which responses were evaluated [15, 16]. Whilst the evaluations were positive, a trinary system does not afford the granularity to elicit response quality in a detailed manner. A study by Muntean et al. [17] evaluated permutations of AMD questions with inputs to the LLM including a contextual scenario that assisted in developing more coherency and appropriate responses. However, the responses to a list of frequently asked AMD related questions input into chatbots without additional context or bias remains a knowledge gap in the field.

In the present study, we assessed the responses from a commonly used LLM, ChatGPT-4, to a list of frequently asked questions in AMD. The evaluations were performed by a team of 10 clinicians from a range of clinical settings to obtain a diverse range of opinions. LLM responses were evaluated using four domains of suitability (coherency, factuality, comprehensiveness, and safety) using a 5-point Likert scale.

## Methods

### Ethics statement

The study adhered to the tenets of the Declaration of Helsinki. Ethics approval was not required as the members of the investigator team reviewed the responses. No other participants were sought.

### Frequently asked questions in AMD

We have previously described this methodology applied to glaucoma [18]. In brief, we first performed a Google search for websites that provided lists of frequently asked questions related to AMD from a patient perspective, including authoritative resources, major health services and networks (including major eye hospitals), and advocacy organisations. These included, but were not limited, to the following major sources: the American Academy of Ophthalmology, American Macular Disease Foundation, Centre for Eye Research Australia (CERA), BrightFocus, Lions Eye Institute, Macular Disease Foundation Australia (MDFA), National Eye Institute, Retina Australia, and others.

Over 200 questions were curated in the initial search. Duplicate or similar questions were combined. The questions were grouped into four themes: 1) definition (of AMD); 2) causes and risk factors; 3) symptoms and detection; and 4) treatment and follow-up. Some questions did not fall into these categories, or were determined to be unanswerable (such as from accuracy or practical perspectives), and were ineligible for inclusion. An example of an ineligible question was “What is the contact number to call if I have an emergency after hours or on weekends?” For this study, we aimed to have general questions broadly applicable across patients with AMD, and so questions related to highly specific drugs or surgical techniques (or were otherwise “low yield” in terms of relevance or specificity; an example was “Which AREDS/AREDS2 formula is right for me?”) were not included in the final list. The final list of questions numbered 37 spread across the four themes. A visual guide to this part of the study is shown in Supplementary Fig. 1.

### Generating Chat-GPT responses

ChatGPT-4 (OpenAI Inc, San Francisco, CA) was used to generate the responses to each question on 14^th^ May 2024. Each question was input into ChatGPT-4, and the response was directly copied and pasted into an external Excel document. No addenda, such as word limits or specific instructions (e.g. “concisely summarise”), were used during each chat session. For each question, a new ChatGPT session was started, i.e. the questions were never asked sequentially within the same session as this could potentially introduce biases into the response. Table 1 shows a list of the questions, and the responses, due to length, are shown in Supplementary Table 1.

**Table 1:** Questions input into ChatGPT-4 to generate responses for evaluation, organised by question theme and number. Questions are written verbatim.

Question theme	Question number	Question input
Definition	1	What is the macula?
	2	What are drusen?
	3	What is age-related macular degeneration?
	4	What is dry age-related macular degeneration?
	5	What is wet age-related macular degeneration?
	6	What are the stages of macular degeneration?
	7	Can the dry form of age-related macular degeneration turn into wet age-related macular degeneration?
	8	If I have drusen, does that mean I have age-related macular degeneration?
Causes and risk factors	9	What causes age-related macular degeneration?
	10	What are the risk factors for age-related macular degeneration?
	11	Is there any way to prevent age-related macular degeneration?
	12	What can I do to reduce my risk of age-related macular degeneration progression?
	13	Are my family members at risk for age-related macular degeneration if I have it?
	14	What causes age-related macular degeneration to get worse?
Symptoms and detection	15	What is an Amsler grid and how do I use it for age-related macular degeneration?
	16	Can age-related macular degeneration lead to blindness?
	17	What are the symptoms of age-related macular degeneration?
	18	How is age-related macular degeneration diagnosed?
	19	How often do I need to get checked for age-related macular degeneration?
	20	How quickly does age-related macular degeneration progress?
	21	Can I still drive if I have age-related macular degeneration?
	22	Can artificial intelligence diagnose age-related macular degeneration?
	23	Do I need the dye injection test to diagnose age-related macular degeneration?
Treatment and follow up	24	How is dry age-related macular degeneration treated?
	25	How is wet-age-related macular degeneration treated?
	26	Is age-related macular degeneration curable?
	27	Do all types of age-related macular degeneration need injections?
	28	Can laser eye surgery treat age-related macular degeneration?
	29	How many treatments do I need for wet age-related macular degeneration?
	30	What are the benefits and how successful is the treatment for wet age-related macular degeneration?
	31	What are the risks and side effects associated with age-related macular degeneration treatment?
	32	What lifestyle factors can help manage my age-related macular degeneration?
	33	Should I take AREDS supplementation for age-related macular degeneration?
	34	What is the difference between AREDS and AREDS2 supplementation for age-related macular degeneration?
	35	Are there any eye drops available for treating age-related macular degeneration?
	36	What is the prognosis for people with age-related macular degeneration?
	37	What can I do if I have already lost some vision from age-related macular degeneration?

### Evaluation of responses

The investigator team represented a cross-section of AMD experts across different professions (optometry and ophthalmology) and settings, including clinical, research and/or teaching responsibilities (*n* = 10). A diverse team was formed to capture a range of opinions on the statements. Prior to evaluating the ChatGPT responses, the investigators were asked to provide their relevant degrees/qualifications, mode(s) of practice, and number of years in clinical practice (Table 2).

**Table 2:** Demographics of the investigator team relevant to the present study.

Years in clinical practice (mean, SD)		11.2 (10.6)
Profession	Optometrist	5
	Ophthalmologist	5
Degrees (*n*)^a^	BOptom or equivalent	6
	Masters	4
	MD or equivalent	5
	PhD	3
Mode of practice (*n*)^a^	Clinical	10
	Teaching	10
	Research	9
	Administration	9
Main mode of practice (*n*)^b^	Clinical	7
	Teaching	2
	Research	2
	Administration	0
Type of work setting (*n*)^a^	Private practice - corporate	1
	Private practice - sole trader/locum	6
	Private practice - not for profit	1
	Secondary referral centre	3
	Hospital – public	5
	Academic institute/university	6

^a^The members of the investigator team often had multiple degrees, modes of practice and worked in different settings; these variables did not necessarily add up to *N* = 12.

^b^One respondent had an equal division in clinical and teaching roles.

The investigators were asked to evaluate the ChatGPT responses across four domains of quality: coherency, factuality, comprehensiveness, and safety, as previously described [18, 19]. Coherency ensures a logical flow of information. Factuality ensures that correct and evidence-based information is provided, and, conversely, that misinformation is avoided. Comprehensiveness reflects enough information to address the question. Safety is important as misleading information could lead to physical and/or psychological harm to patients. In this study, each domain was represented by a separate question and was evaluated independently, i.e. a statement could be coherent but not factual.

A 5-point Likert scale was used for the evaluations, with a higher number indicating higher quality in relation to the domain (1 represented strong disagreement (e.g. strongly disagreeing that the statement was coherent) and 5 represented strong agreement with the statements). A 5-point Likert scale was used to provide more granularity than a 3-point scale, whereby “2” (“disagree”) or “4” (“agree”) could provide further insights into raters’ opinions, such as having room for improvement or not being completely erroneous. Scores “1” and “5” also provide the avenue for more “extreme” views. We chose 5 points (rather than more) as it offers a balance between time taken for grading alongside reliability, validity and discriminating power [20]. There were free-text comment boxes for respondents to add comments in relation to their evaluations.

We did not introduce any further prompts and simply asked raters whether a statement was coherent, factual, comprehensive or safe from their perspective. We did not ask raters to use specific clinical guidelines or reference specific evidence from the literature, but instead relied on their own training and perspectives, as doing so may unwittingly homogenise the responses. The invitation for free-text comments provided an avenue for justification for ratings, where relevant. Finally, we did not specify to the rater that one or more domains were considered to be more important, as the goal was to assess each quality domain independently.

Each investigator performed the evaluations independently. The responses were captured on an online platform and exported into a separate Excel file.

### Statistical analysis

The analysis approach was similar to our previous work [18]. For descriptive statistics, we reported median and mode reflected the overall frequency of scores, as reported by previous work in the field [15, 16]. We also reported mean and standard deviation (SD) to examine for subtle differences between themes and quality domains, as median may lack granularity. Multiple comparisons were performed between domains (one-way ANOVA with a main effect of domain) and rater groups (paired t-tests). Specifically, we were interested in whether domains were rated differently, and therefore whether specific improvements to chatbot outputs could be targeted. Due to the unequal number of questions across domains, a main effect of question was not used for the above comparisons. Inter-rater agreement was assessed using Fleiss’s kappa (on the 5-point Likert scale and on a 3-point disagree-neither-agree scale). Fleiss’s kappa was not accompanied by an interpretation, since the guidelines suggested by Landis and Koch [21] refer specifically to a 2-annotator, 2-outcome analysis. Cronbach’s alpha was used to evaluate the reliability of the questions. A *p* < 0.05 was considered statistically significant, following Bonferroni correction for multiple comparisons. Analyses were performed on GraphPad Prism version 8 (La Jolla, CA) and Microsoft Excel version 2408 (Microsoft Corporation, Redmond, WA).

## Results

### Evaluators’ scores

The descriptive statistics for overall scores across the themes and domains are summarised in Table 3 (and presented in Supplementary Figs. 2–5). Median scores across themes and domains were all 4 (corresponding to “agree”). A Likert score of 4 was also the most common result across all questions and evaluations. Comprehensiveness had the lowest scores across domains (mean 3.8 ± 0.8), followed by factuality (mean 3.9 ± 0.8), safety (mean 4.1 ± 0.8), and finally coherence (mean 4.3 ± 0.7).

**Table 3:** Summary descriptive statistics of Likert scores across question themes and quality domains.

	Definition			Causes and risk factors			Symptoms and detection			Treatment and follow up		
Quality domains	Mean (SD)	Median (IQR)	Mode	Mean (SD)	Median (IQR)	Mode	Mean (SD)	Median (IQR)	Mode	Mean (SD)	Median (IQR)	Mode
Coherency	4.4 (0.6)	4 (4,5)	4	4.3 (0.7)	4 (4,5)	4	4.2 (0.7)	4 (4,5)	4	4.2 (0.8)	4 (4,5)	4
Factuality	3.9 (0.8)	4 (4,4.3)	4	3.9 (0.8)	4 (3,4)	4	3.8 (0.8)	4 (3,4)	4	3.9 (0.9)	4 (4,4)	4
Comprehensiveness	3.8 (0.9)	4 (3,4)	4	4.1 (0.6)	4 (4,4)	4	3.9 (0.9)	4 (4,4)	4	3.8 (0.8)	4 (3,4)	4
Safety	4.2 (0.7)	4 (4,5)	4	4.2 (0.6)	4 (4,5)	4	4.1 (0.8)	4 (4,5)	4	4.1 (0.9)	4 (4,5)	4

*IQR* interquartile range, *SD* standard deviation.

We examined the number of questions that had an average Likert score below 4 (i.e. below “agree”). For coherency, the fewest number of questions met this criterion at 5/37 (14%, Supplementary Fig. 2). For factuality and comprehensiveness, over half of questions averaged a score below 4, at 21/37 (57%, Supplementary Fig. 3) and 23/37 (62%, Supplementary Fig. 4). The safety domain had the second fewest number of questions meeting this criterion at 9/37 (24%, Supplementary Fig. 5). The differences in average score across the domains was statistically significant (*p* < 0.0001), which persisted for all multiple comparisons, except when comparing factuality and comprehensiveness, for which there was no statistically significant difference (*p* = 0.9801).

Multiple comparisons showed that the differences between coherency and factuality, coherency and comprehensiveness, factuality and safety, and comprehensiveness and safety were significant for most of the domains (*p* = 0.0399 to <0.0001). The differences between coherency and safety (*p* = 0.1192–0.5181), and factuality and comprehensiveness (*p* = 0.3613–0.9213) were not significant across all domains.

### Evaluators’ comments to ChatGPT responses

Questions that were, on average, rated at a Likert score <4 across three or more domains underwent further analysis of any associated free-text comments from the raters. Nine out of 37 questions (24%) met this criterion. Comments in response to these questions are shown in Supplementary Table 2. There were several consistent themes arising from the raters’ comments.

In response to question 13, five raters explicitly identified the lack of utility of genetic testing for AMD. Similarly, in response to question 18, raters did not advocate for genetic testing for AMD. Additionally, raters identified fluorescein angiography (FA) and indocyanine green (ICG) as non-routine testing in early AMD. In response to question 19, raters highlighted the prescriptive and vague nature of the review periods, suggesting that a more tailored approach, such as by assessing risk and treatment, is required. Responses to question 21 was rated poorly, with the advice being deemed unsuitable due to the potential diversity of visual function possible in AMD. Responses to question 24 were also mixed, with criticisms directly at the responses appearing to pivot to wet/neovascular AMD, which means its advice was not suitable. Furthermore, several advances in dry/atrophic AMD treatments were not stated (similar comments were made for the responses to questions 27 and 28). Raters consistently identified that surgical options in response to question 26 were not suitable in this context. Similarly, the mention of miniature telescopes was identified as infrequent clinical practice and were suggested to be removed (question 31).

As described above, thematically, the factuality and comprehensive domains were most commonly-rated low for the above subset of questions, and the comments reflected this deficit. Overall, several statements appeared to be out-of-date, missing out on more recent clinical practice patterns and novel treatments. Conversely, older or more niche practices were stated in responses, potentially surpassed by the adoption of newer technologies, such as OCT-A.

### Inter-rater agreement between responses and internal reliability of questions

Using the 5-point Likert scale, Fleiss’s kappa was lowest for factuality (0.351) and was highest for coherency (0.409) (Table 4). Kappa values were higher when using a 3-point scale (where strongly disagree and disagree were combined into one variable, and similarly for agree and strongly disagree), where comprehensiveness was the lowest (0.560), and coherency remaining the highest (0.831). Cronbach’s alpha values were generally high across all four domains. The domain with the highest internal reliability was coherency (0.958), and the lowest was factuality (0.911).

**Table 4:** Fleiss’s kappa values for each domain when using a 5-point Likert scale and when the scale was condensed into a 3-point scale (disagree-neither-agree).

	Fleiss’s kappa (5-point scale)	Fleiss’s kappa (3-point scale)	Cronbach’s alpha
Coherency	0.409	0.831	0.958
Factuality	0.351	0.586	0.911
Comprehensiveness	0.370	0.560	0.951
Safety	0.359	0.718	0.957

### Differences in scores between optometrists and ophthalmologists

**Fig. 1:**
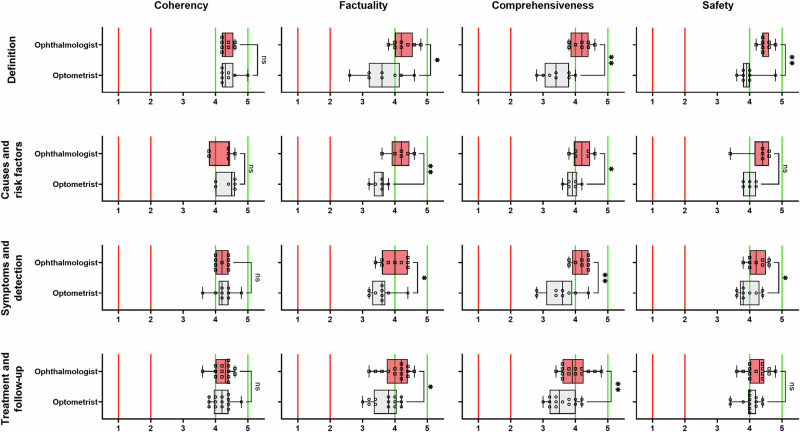
Distribution of Likert scores for each theme (rows) and quality domain (columns) when separated by professional group (ophthalmologist, squares and red bars; optometrist, circles and grey bars). The vertical red solid line indicates Likert scores 1 and 2 (unfavoured, “negative” results) and the green solid line indicates Likert scores 4 and 5 (favoured, “positive” results). The vertical brackets indicate levels of significance when comparing professional groups (ns indicates not statistically significant; *p < 0.05; **p < 0.01).

Comparisons between the two professional groups across each theme and domain are shown in Fig. 1. There were no significant differences between groups when considering coherency across all themes (*p* = 0.2664–0.6224), and for safety across the causes and risk factors and treatment and follow-up themes (*p* = 0.6034–0.1780). There were significant differences across the other conditions, with the optometrist group rating the responses lower compared to the ophthalmologist group (*p* = 0.0446–0.0010).

## Discussion

A diverse group of eye clinicians evaluated ChatGPT-4 responses to frequently asked questions regarding AMD as coherent, factual, comprehensive and safe. The coherency domain was evaluated highest, followed by safety, factuality and comprehensiveness. Whilst the Likert scores were generally agreeable at the group level, evaluator ratings were variable, with over half of the questions scoring below 4 within the factuality and comprehensiveness domains. Free-text comments identified areas of deficit, and a substantial number of questions scored below an “agree” level across key quality domains, especially with respect to factuality, specificity and applicability of the information, and its contextualisation.

### Performance of ChatGPT-4 in responding to frequently asked questions in AMD

Ferro Desideri et al. [15] compared the three LLMs in answering general medical advice (15 questions) and advice related to intravitreal injections (13 questions) for AMD and used three retina specialists to assess their accuracy and sufficiency (comprehensiveness). Specific to ChatGPT performance, the authors found that 12/15 responses to general medical advice questions were deemed accurate and sufficient, and the other three responses were partially accurate and sufficient. For the questions related to intravitreal injections, 10/13 responses were deemed accurate and sufficient, and three were partially accurate and sufficient. These results suggested an optimistic view of LLM responses. However, their study did not report on what characteristics of the responses were deemed to only be partially accurate. Furthermore, although the authors reported a high level of sufficiency of responses (analogous to comprehensiveness in our present work), our results demonstrated lower ratings in this quality domain. The methodological approach also differed, as our study used a Likert scale, which provides more granularity than their trinarised, descriptive rating. As stated in the Methods, a 5-point scale permits the expression of more “extreme” views and more tempered opinions (and thus granularity), relative to a 3-point scale, whilst maintaining more efficiency and potentially better test-retest reliability compared to bigger scales, such as a 10-point scale [20].

Cheong et al. [16] evaluated the responses of several chatbots, including ChatGPT-4, to questions related to the macula and retina. Three fellowship-trained retinal specialists evaluated the chatbot responses using a 3-point Likert scale (0–2) and summed the scores across the graders to reflect a consensus approach to evaluation. They found that 83.3% of ChatGPT-4’s responses to the AMD questions were “good” (their highest rating), with none of the responses deemed “poor” (their lowest rating). ChatGPT-4 (and 3.5) outperformed the other chatbots in the study, and the authors concluded that they are potentially capable of answering questions related to retinal diseases, such as AMD. Differences between our present study and the work of Cheong et al. [16] included the scope of questions, and the method of grading. Their list of AMD questions was mostly thematically related to treatment and associated advice, such as vitamins and processes related to intravitreal injections, with some questions being highly specific (such as a question related to verteporfin (Visudyne, Bausch and Lomb, Ontario, Canada) and ranibizumab (Lucentis, Novartis AG, Basel, Switzerland); notably, the chatbot specifically used trade names, rather than the generic term). While their consensus approach was useful to obtain an overall impression of quality, it did not facilitate analysis of the variability across graders.

Muntean et al. [17] conducted a study comparing ChatGPT-4, PaLM2 and three ophthalmologists’ responses to specific scenario questions, incorporating a background vignette (such as that asker of the question is a patient with AMD) that may be relevant to formulating the result. Using these permutations, the authors analysed the results of 133 questions along six axes of quality, some of which overlapped with our quality domains. Using two ophthalmologist reviewers, the authors reported very positive results for ChatGPT 4 responses, with 88–100% of responses obtaining a perfect score of 5 (on a 5-point Likert scale) which were higher compared to our results. Key differences between their methodology and the present study could explain the differences in results. One difference was the comprehensiveness of the system and user prompts input by Muntean et al. [17], which includes several important caveats, two of which were to ask the chatbot to explain why a question may not make sense instead of answering a confusing or incorrect question, and to not share false information if the chatbot does not know the answer. There were many instances in the present study where the information was not accurate or relevant to the question, which could be addressed by the inclusion of these prompts. Prefacing and contextualising the question could assist in provide more relevant and safe advice in the responses. Despite the optimism across most of the quality domains, Muntean et al. [17] also highlight the deficits related to the responses in terms of their reflection of clinical and scientific consensus (i.e. contemporaneous and correct medical knowledge) and not missing important information, similar to the criticisms raised in our results.

Overall, previous literature related to chatbot usage in AMD has been mostly positive, especially regarding the accuracy and comprehensiveness of responses. However, our study was comparatively less positive, possibly due to a greater diversity of graders, a wider range of questions and the use of a 5-point Likert scale across more domains of quality. Unsurprisingly, whilst coherency was the top-rated domain, its importance is arguably lower than that of safety and factuality, as these reflect the potential risks to the community with unsupervised chatbot use.

### Variability in evaluations amongst evaluators and by professional group

The diverse team of raters in the present study indicates that the accuracy or utility of chatbots may differ depending on clinical setting and the patient base. For example, general optometric practices are more likely to see patients at risk of AMD or with earlier stages of AMD. Conversely, specialist ophthalmology clinics are more likely to see patients with more advanced stages of AMD and those requiring treatments, such as intravitreal injections. Other specific services, such as low vision clinics and collaborative care settings, may also impact the patient base and information expected from the chatbot [22, 23].

The optometrist group returned lower ratings in comparison to the ophthalmologist group. One explanation for this may be the more conservative attitude of the optometrist group, which comprised clinicians working in a primarily academic setting. Criticisms related to comprehensiveness of chatbot responses may reflect a professional habit of covering more information and content, given more attendance time by the professional group. The academic clinical setting may reflect a more critical attitude of the optometrist group in the present study, seeking more precise language regarding chatbot outputs.

Another explanation is the potential heterogeneity amongst all raters, and the acceptability of different levels of precision of chatbot statements. Although there are guidelines for care of patients with AMD [24–26], differences at the professional level may also inject biases into interpretation of chatbot outputs. Despite authoritative guidelines, it is also known that consensus on statements regarding AMD within and between professions may be difficult to achieve, due to the wide heterogeneity of clinical practices and patient presentation [27].

### Separating quality domains in evaluating chatbot responses

Coherency being rated highest was expected, given the nature of LLM chatbot technology [28]. This domain of chatbot response quality tends to be highly rated within the literature across many fields. One notable issue was the lack of citations in some of the responses [29].

Safety-wise, a feature of many of the responses were recommendations for seeking expert advice from an eye care professional. This was particularly important for the treatment themed questions. However, several questions were rated poorly in safety for other reasons, most notably due to poor advice regarding unnecessary tests or interventions. An example that was repeatedly criticised was genetic testing, which, at the time of the study, is not a routine clinical test for AMD [30].

Factuality also had many questions with suboptimal ratings. An issue that was raised by Muntean et al. [17] was the role of system prompts to ensure an appropriate answer, and the responses to our approach further highlighted flaws of information saliency. Several of the chatbot responses may have been strictly true, but were far removed from routine clinical practice, and the lack of prioritisation of important information meant that the facts were not accurately represented.

The problem of information saliency was also reflected in low comprehensiveness scores. The chatbot responses would sometimes include niche information, such as low vision aids and telescopes. Muntean et al. [17] attempted to pre-empt this limitation by adding a patient’s scenario to preface the question. However, again, a layperson using LLM technology may not have the expertise to add this information to optimise the response. A limitation of pre-trained LLMs is the potential dated information, where emerging technologies and treatments cannot be included in the responses.

### Limitations

We have previously described the limitations of the subjective rating approach to evaluating LLM responses [18]. Combinations of multi-point Likert or other granular scales and having more graders may help to overcoming skewed subjective data. Although 5-point Likert scales are more granular than trinary scales, there is still the potential for ceiling or floor effects [31]. This was seen with many of the questions having a score of 4 or greater. Studies of this nature also lack a ground truth, instead relying on validity determined by experts. Reference standards are available when comparing across different LLMs or expert human-generated outputs, but these also have issues with subjectivity.

Our list of questions was curated from several authoritative sources, and were, in large part, simplified for the purposes of brevity. As described above, how questions are input into chatbots may contribute to response generation. Our goal was to keep the questions simple and broad. Future studies with more granularity could provide further insights.

Finally, to further understand clinical implementation would require end-user input, such as patients at risk of or who have AMD. Alongside further stakeholder consultation, there are well documented ethical challenges occurring in parallel to clinical issues of accuracy, with many concerns such as privacy and security, intellectual property, transparency and accountability, bias and explainability, amongst others [32]. This is another consideration for clinicians prior to widespread deployment.

## Conclusions

ChatGPT-4 provides responses to frequently asked questions in AMD, with generally agreeable ratings of coherency, factuality, comprehensiveness, and safety. However, there were key areas of weakness, including notable omissions in more recent advances in the field, thereby potentially misleading patients with out-of-date facts and raising safety issues with unsupervised use. These results suggest that routine unsupervised use of ChatGPT-4 in a patient facing setting is not advised at this stage.

## Summary

### What was known before

- Chatbots are potentially useful adjunctive tools to assist clinicians in answering patient questions.
- Chatbots may some useful answers to frequently asked questions regarding age-related macular degeneration.

### What this study adds

- ChatGPT-4, without additional curated prompting, provides modest quality responses (across domains of coherency, factuality, comprehensiveness, and safety) to frequently asked questions regarding age-related macular degeneration.
- Key weaknesses of ChatGPT-4 responses include notable recent omissions in the field of age-related macular degeneration and reporting of obsolete information.

## Supplementary information

Supplementary Figure Captions

Supplementary Figure 1

Supplementary Figure 2

Supplementary Figure 3

Supplementary Figure 4

Supplementary Figure 5

Supplementary Table 1

Supplementary Table 2

## Data Availability

All data are available from the corresponding author upon a reasonable request.
