# Supplementary material for: ChatGPT-4 for addressing patient-centred frequently asked questions in age-related macular degeneration clinical practice
Source: Eye (Lond). 2025 Apr 15;39(10):2023–30. doi: 10.1038/s41433-025-03788-0 (PMC12209409; doi:10.1038/s41433-025-03788-0)
Supplement: Supplementary file 1 — Supplementary Figure Captions [file 41433_2025_3788_MOESM1_ESM.docx]

**Supplementary Material: ChatGPT-4 for addressing patient-centred frequently asked questions in age-related macular degeneration clinical practice**

Henrietta Wang^1,2^, Amanda Ie^3,4^, Thomas Chan^3,5^, William Yates^6^, Michael Kalloniatis^1,7,8^, Janelle Tong^1,2^, Sophia Zhang^1,2^, Tracey Phan^9^, Christopher Go^6,10,11^, Jack Phu^1,2,12,13^

1. School of Optometry and Vision Science, University of New South Wales, Kensington, New South Wales, Australia
2. Centre for Eye Health, University of New South Wales, Kensington, New South Wales, Australia
3. Department of Ophthalmology, Westmead Hospital, Westmead, NSW, Australia
4. Department of Ophthalmology, Westmead Children’s Hospital, Westmead, NSW, Australia
5. Discipline of Ophthalmology and Eye Health, Faculty of Medicine and Health, University of Sydney, NSW, Australia
6. Save Sight Institute, Faculty of Medicine and Health, University of Sydney, NSW, Australia
7. School of Medicine (Optometry), Deakin University, Waurn Ponds, Victoria, Australia
8. University of Houston College of Optometry, Houston, Texas, USA
9. Department of Ophthalmology, Liverpool Hospital, Liverpool, NSW, Australia
10. School of Clinical Medicine, Faculty of Medicine and Health, University of Sydney, Sydney, NSW, Australia
11. Vision Eye Institute, NSW, Australia
12. Faculty of Medicine and Health, University of Sydney, Sydney, NSW, Australia
13. Concord Clinical School, Concord Repatriation General Hospital, Concord, NSW, Australia

Number of Supplementary Figures: 4

Number of Supplementary Tables: 2

Corresponding Author: Jack Phu

Address for reprints: School of Optometry and Vision Science, Gate 14 Barker Street Rupert Myers Building South Wing, University of New South Wales Sydney 2052, New South Wales, Australia

Email: [jack.phu@unsw.edu.au](mailto:jack.phu@unsw.edu.au)

*Keywords*: large language models; artificial intelligence; chatbot; conversation agents; collaborative care; macular disease; macular degeneration; OCT; optical coherence tomography; fundus autofluorescence

Financial support: The work was supported, in part, by an NHMRC Ideas Grant to MK and JP (1186915). The funding organisation had no role in the design or conduct of this research.

Conflict of interest: No conflicting relationship exists for any author.

Running head: ChatGPT-4 in AMD

*Supplementary Figure 1: Flowchart showing the curation of the initial pool of 203 questions into the final list of 37 questions examined in the present study.*

*Supplementary Figure 2: Distribution of Likert scores for each question for the coherency quality domain. The solid black vertical lines and bars indicate the median and interquartile range, respectively. The thin horizontal lines indicate the full range of scores. Open circles indicate a response from an evaluator. The small plus sign (+) indicates the mean. The vertical red solid line indicates Likert scores 1 and 2 (unfavoured, “negative” results) and the green solid line indicates Likert scores 4 and 5 (favoured, “positive” results). The horizontal filled bars have been coloured according to their question theme (grey: definitions, e.g. “What is age-related macular degeneration?”; yellow: causes and risk factors, e.g. “What causes age-related macular degeneration?”; blue, symptoms and detection, e.g. “What is an Amsler grid and how do I use it for age-related macular degeneration?”; red, treatment and follow up, e.g. “How is wet age-related macular degeneration treated?”).*

*Supplementary Figure 3: Distribution of Likert scores for each question for the factuality quality domain. All other details as per* *Supplementary Figure 2.*

*Supplementary Figure 4: Distribution of Likert scores for each question for the comprehensiveness quality domain. All other details as per Supplementary Figure 2.*

*Supplementary Figure 5: Distribution of Likert scores for each question for the safety quality domain. All other details as per Supplementary Figure 2.*
