# Supplementary figures and images for: ChatGPT-4 for addressing patient-centred frequently asked questions in age-related macular degeneration clinical practice
Source: Eye (Lond). 2025 Apr 15;39(10):2023–30. doi: 10.1038/s41433-025-03788-0 (PMC12209409; doi:10.1038/s41433-025-03788-0)

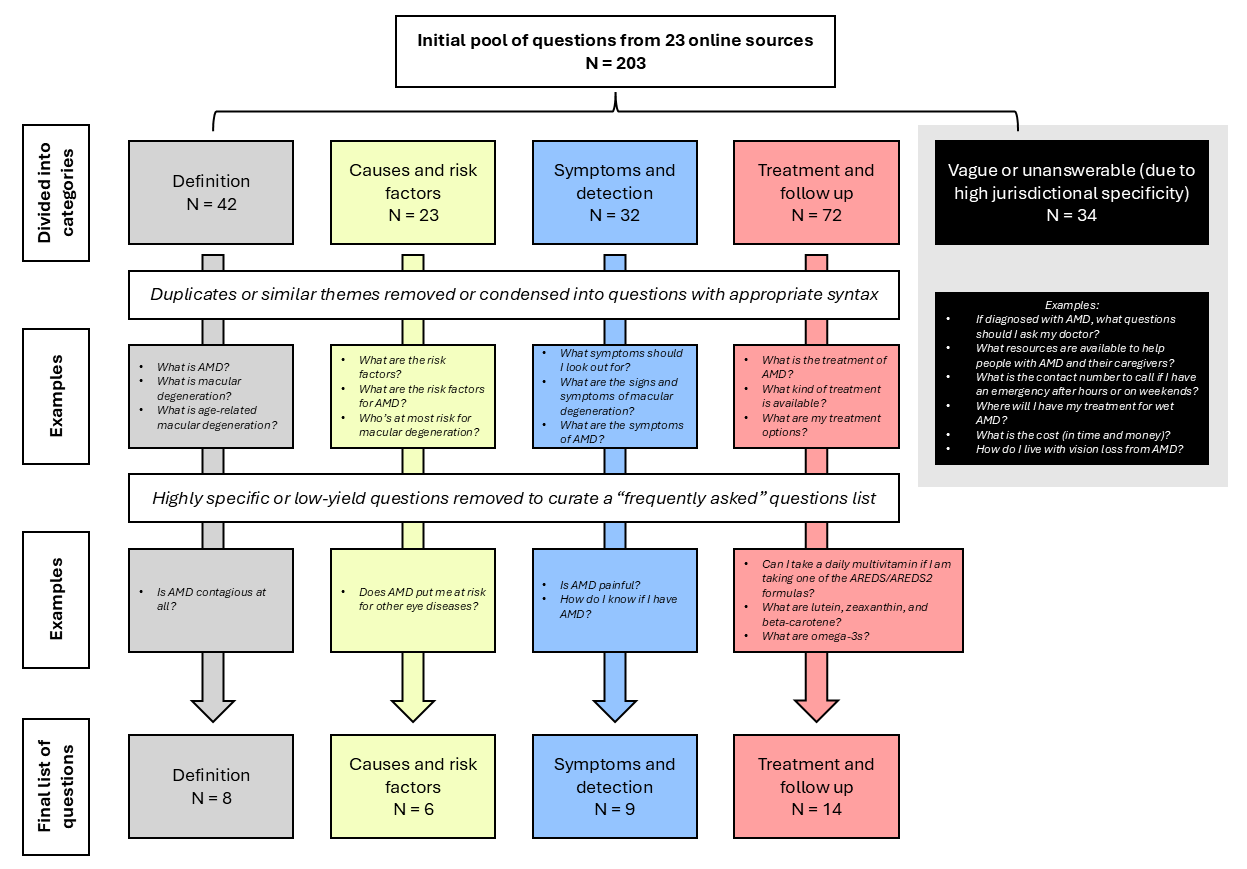

Supplement: Supplementary file 2 — Supplementary Figure 1 [file 41433_2025_3788_MOESM2_ESM.tif]

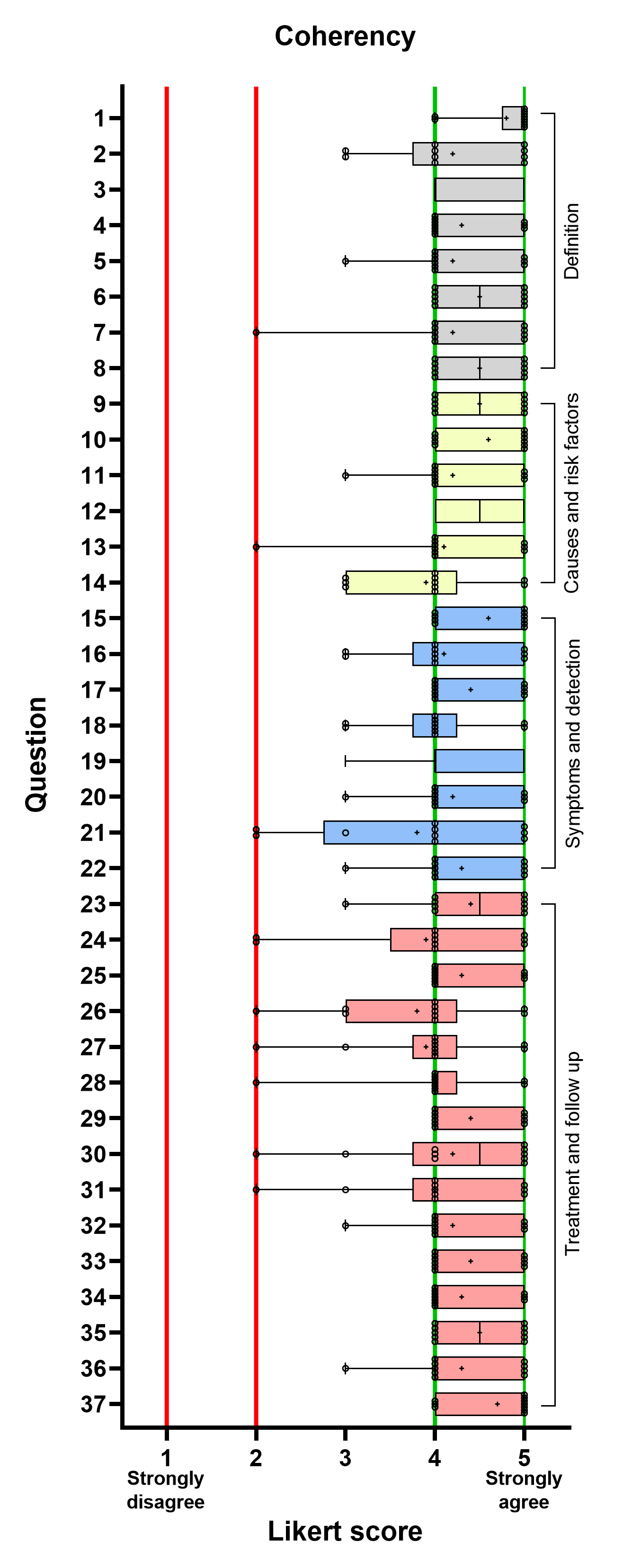

Supplement: Supplementary file 3 — Supplementary Figure 2 [file 41433_2025_3788_MOESM3_ESM.tif]

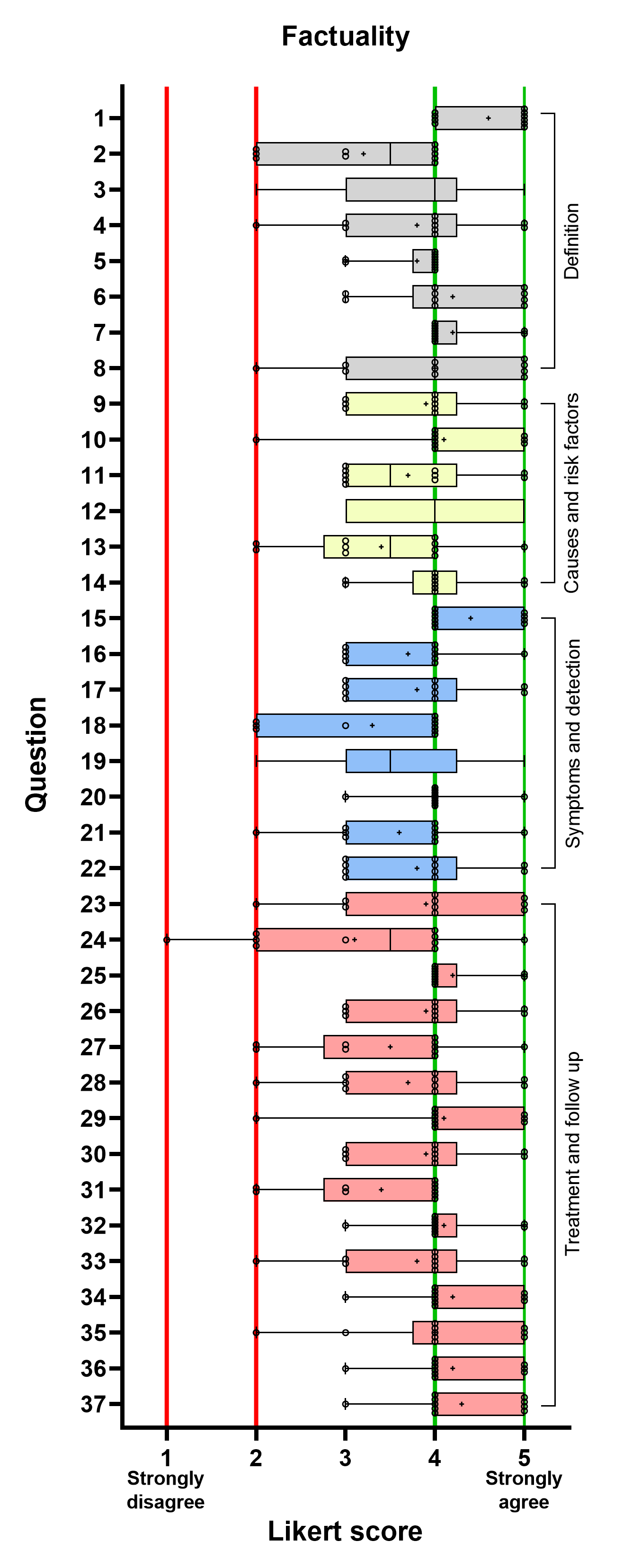

Supplement: Supplementary file 4 — Supplementary Figure 3 [file 41433_2025_3788_MOESM4_ESM.tif]

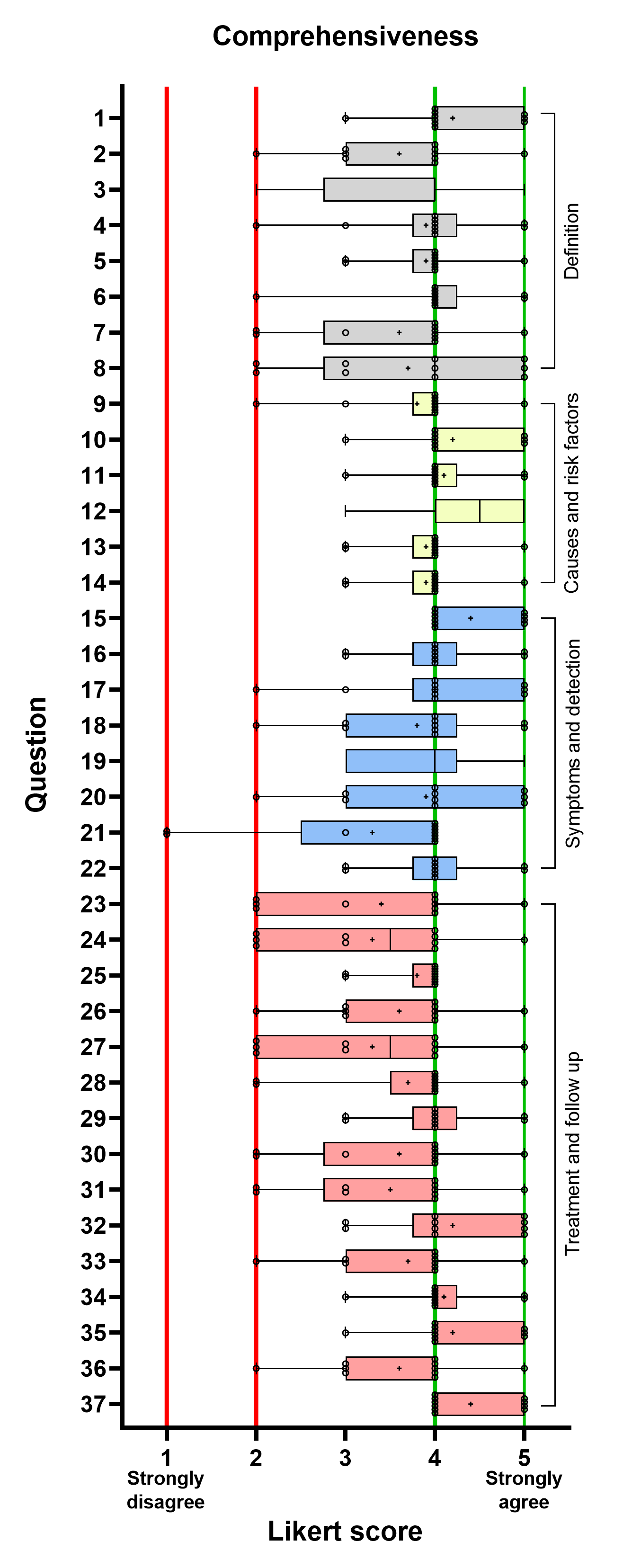

Supplement: Supplementary file 5 — Supplementary Figure 4 [file 41433_2025_3788_MOESM5_ESM.tif]

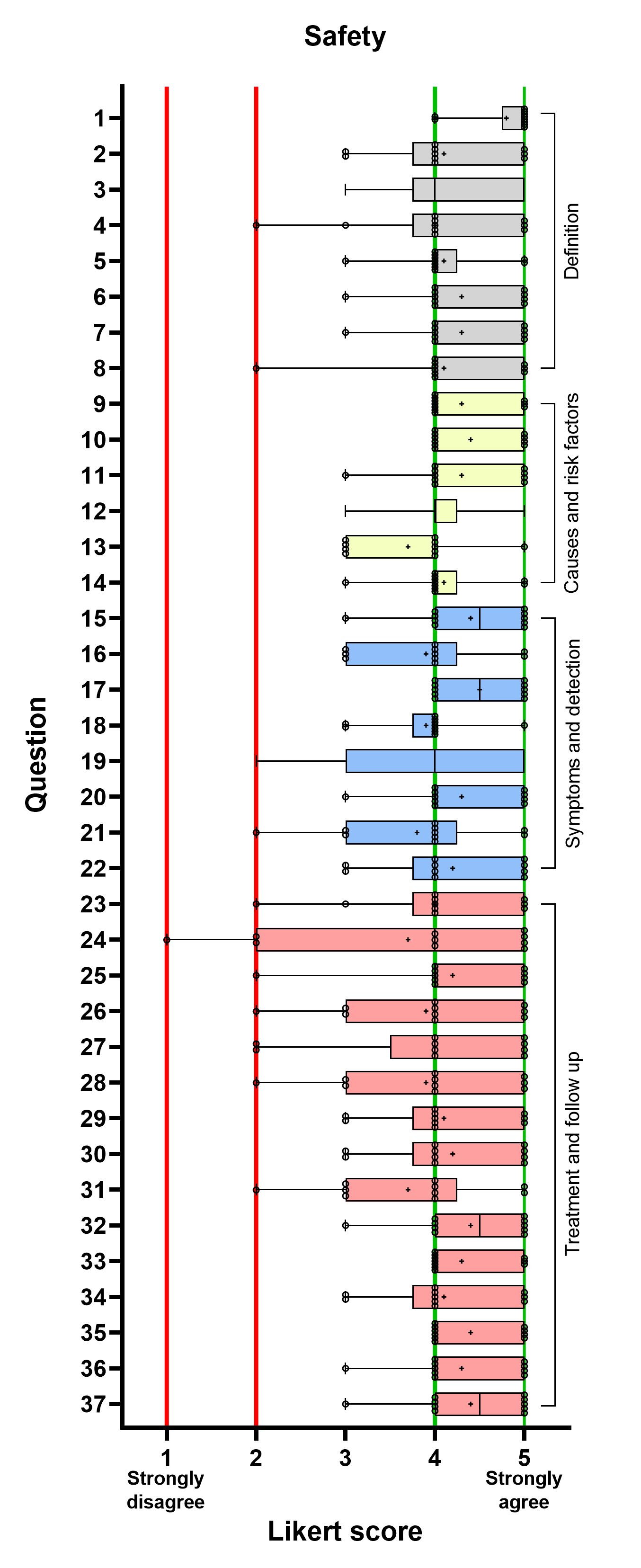

Supplement: Supplementary file 6 — Supplementary Figure 5 [file 41433_2025_3788_MOESM6_ESM.tif]
