# Supplementary material for: ChatGPT-4 for addressing patient-centred frequently asked questions in age-related macular degeneration clinical practice
Source: Eye (Lond). 2025 Apr 15;39(10):2023–30. doi: 10.1038/s41433-025-03788-0 (PMC12209409; doi:10.1038/s41433-025-03788-0)
Supplement: Supplementary file 7 — Supplementary Table 1 [file 41433_2025_3788_MOESM7_ESM.docx]

| Supplementary Table 1: Question inputs and ChatGPT-4 outputs as described in the Methods. Inputs and outputs are shown verbatim. For visibility, the cells have been colour coded according to each of the four themes as demonstrated in Figures 1-4 of the main text. | | | |
| --- | --- | --- | --- |
| Question theme | Question number | Question input | ChatGPT-4 output |
| Definition | 1 | What is the macula? | The macula is a small, specialized area in the center of the retina, which is the light-sensitive tissue at the back of the eye. It is responsible for central vision, allowing us to see fine details clearly. The macula contains a high concentration of cone cells, which are photoreceptor cells that are particularly sensitive to light and responsible for color vision. The macula is crucial for activities such as reading, recognizing faces, and driving. It plays a key role in our ability to focus on and perceive objects in the central part of our visual field. Conditions such as age-related macular degeneration (AMD) can affect the macula and lead to vision impairment, especially in older individuals. |
|  | 2 | What are drusen? | Drusen are small yellow or white deposits that accumulate under the retina of the eye. They are a common feature of aging and are often associated with age-related macular degeneration (AMD), which is a leading cause of vision loss in older adults.  Drusen are composed of a variety of materials, including lipids, proteins, and cellular waste products. They can be categorized into two main types: "hard" drusen and "soft" drusen. 1. **Hard Drusen:** These are small, discrete, and usually less than 63 micrometers in size. Hard drusen are considered a normal part of aging and may not necessarily lead to vision problems. However, the presence of a large number of hard drusen or certain characteristics may increase the risk of developing AMD. 2. **Soft Drusen:** These are larger and more diffuse than hard drusen. Soft drusen are associated with the progression of AMD. As AMD advances, the retinal pigment epithelium (RPE) can be damaged, leading to vision loss. The accumulation of drusen in the retina is a complex process, and the exact cause of AMD is not fully understood. However, risk factors for AMD include age, genetics, smoking, and certain genetic variations. Regular eye exams are essential for detecting the presence and characteristics of drusen, as well as monitoring changes in the retina that may indicate the progression of AMD. Early detection and management are crucial for preserving vision in individuals with AMD. If you have concerns about your eye health, it is recommended to consult with an eye care professional for a comprehensive eye examination. |
|  | 3 | What is age-related macular degeneration? | Age-related macular degeneration (AMD) is a progressive eye condition that primarily affects individuals as they age. It is a leading cause of vision loss among people aged 50 and older. AMD specifically affects the macula, which is the central part of the retina responsible for sharp, central vision needed for activities like reading, driving, and recognizing faces.  There are two main types of AMD: 1. **Dry AMD (Non-neovascular AMD):** This is the more common form, accounting for about 80-90% of AMD cases. It involves the gradual breakdown of light-sensitive cells in the macula, leading to a slow loss of central vision. Dry AMD is characterized by the presence of small yellow deposits called drusen under the retina.  2. **Wet AMD (Neovascular AMD):** Although less common, wet AMD is more severe. It occurs when abnormal blood vessels grow underneath the retina. These new vessels are fragile and can leak blood and fluid, causing damage to the macula. Wet AMD can lead to a more rapid and severe loss of central vision compared to dry AMD. The exact cause of AMD is not fully understood, but age, genetics, and environmental factors such as smoking are known to increase the risk. There is currently no cure for AMD, but early detection and management can help slow the progression of the disease and preserve vision. Treatment options for wet AMD may include anti-VEGF drugs, which help reduce the growth of abnormal blood vessels. Regular eye exams are crucial for early detection and management of age-related macular degeneration. |
|  | 4 | What is dry age-related macular degeneration? | Age-related macular degeneration (AMD) is a condition that affects the macula, a small but critical part of the retina at the back of the eye. The macula is responsible for central vision, which is essential for activities such as reading, driving, and recognizing faces. There are two main types of age-related macular degeneration: dry (atrophic) AMD and wet (neovascular) AMD. Dry AMD is the more common form, accounting for about 80-90% of all AMD cases. In dry AMD, small yellow deposits known as drusen accumulate in the macula. Drusen are extracellular deposits that can interfere with the normal functioning of the retina, leading to a gradual breakdown of light-sensitive cells and supporting tissues in the macula. As the disease progresses, individuals with dry AMD may experience a gradual loss of central vision. Dry AMD typically develops slowly and may progress over several years. It can affect one or both eyes. While there is currently no cure for dry AMD, there are treatments and interventions that may help manage the condition and slow its progression, such as dietary supplements and lifestyle modifications. It's important for individuals experiencing changes in their vision, especially in the central visual field, to seek professional eye care and undergo regular eye examinations for early detection and management of AMD. Wet AMD, the less common but more severe form, involves the growth of abnormal blood vessels beneath the retina and is associated with more rapid vision loss. |
|  | 5 | What is wet age-related macular degeneration? | Wet age-related macular degeneration (wet AMD) is a medical condition that affects the macula, a small but crucial part of the retina at the back of the eye. The macula is responsible for central vision, allowing you to see fine details clearly. AMD is a common eye disorder, particularly in older adults.  There are two main types of age-related macular degeneration: dry AMD and wet AMD. Wet AMD is less common but generally more severe than dry AMD. In wet AMD, abnormal blood vessels grow beneath the macula and leak fluid and blood, leading to damage and distortion of the central vision. This can result in a rapid and severe loss of central vision.  Symptoms of wet AMD may include distorted or blurred vision, a dark spot in the central vision, or difficulties recognizing faces and details. Straight lines may appear wavy, and colors may seem less vibrant.  Wet AMD requires prompt medical attention, as it can progress rapidly and cause significant vision loss. Treatment options may include injections of medications into the eye to inhibit the growth of abnormal blood vessels or laser therapy. It's important for individuals experiencing symptoms or at risk for AMD to have regular eye exams to detect and address any issues early on. |
|  | 6 | What are the stages of macular degeneration? | Macular degeneration, also known as age-related macular degeneration (AMD), is a progressive eye condition that affects the macula, a small area in the retina responsible for central vision. There are two main types of macular degeneration: dry AMD and wet AMD. The progression of the disease can be categorized into different stages. It's important to note that symptoms and progression can vary among individuals. The stages are typically identified through a comprehensive eye examination, including imaging tests like optical coherence tomography (OCT) and fundus photography. The stages are as follows:  1. **Early AMD:**  - Often asymptomatic or with minimal symptoms.  - Drusen, small yellow or white deposits, may be present on the retina but are usually not noticeable without an eye exam.  2. **Intermediate AMD:**  - More noticeable vision changes may occur.  - Larger drusen or pigment changes in the macula.  - Some individuals may experience blurred or distorted central vision.  3. **Late AMD (Dry AMD):**  - Advanced stage of the disease.  - Severe vision loss may occur due to extensive damage to the macula.  - Drusen may have grown in size and number.  - Geographic atrophy, the loss of retinal cells, may be present.  4. **Late AMD (Wet AMD):**  - In wet AMD, abnormal blood vessels grow beneath the retina and leak blood and fluid.  - Sudden and severe vision loss can occur.  - Distorted or blurred vision may result from bleeding or fluid accumulation in the macula.  It's important to note that not all cases of dry AMD progress to the wet form, and the rate of progression varies among individuals. Regular eye exams and early detection are crucial for managing macular degeneration and preserving vision. There is currently no cure for AMD, but certain treatments, such as anti-VEGF medications, can help slow the progression of wet AMD and, in some cases, improve vision. Lifestyle modifications, including a healthy diet, not smoking, and protecting the eyes from UV light, may also be recommended to reduce the risk of progression. If you suspect you may have macular degeneration or are at risk, it's essential to consult with an eye care professional for proper evaluation and guidance. |
|  | 7 | Can the dry form of age-related macular degeneration turn into wet age-related macular degeneration? | Age-related macular degeneration (AMD) is generally classified into two main types: dry AMD (also called non-neovascular or atrophic AMD) and wet AMD (also called neovascular or exudative AMD). These types refer to the different forms of the disease based on the characteristics of the changes in the macula, which is the central part of the retina responsible for sharp, central vision.  Dry AMD is more common and typically progresses more slowly than wet AMD. In dry AMD, cellular debris called drusen accumulates in the macula, leading to the gradual breakdown of light-sensitive cells in the retina. While dry AMD can cause vision loss, it does not involve the abnormal growth of blood vessels characteristic of wet AMD.  Wet AMD, on the other hand, is characterized by the growth of abnormal blood vessels beneath the retina. These vessels can leak fluid and blood, causing rapid and severe damage to the macula, leading to more sudden and significant vision loss.  It's important to note that dry AMD can sometimes progress to wet AMD. This progression occurs in a subset of individuals, and not everyone with dry AMD will develop the wet form. Regular eye examinations and monitoring are crucial for detecting any changes in the condition. Early detection and intervention are key in managing wet AMD, as certain treatments are available to help slow down the progression and, in some cases, improve vision.  If you or someone you know is experiencing changes in vision or has been diagnosed with AMD, it's essential to consult with an eye care professional for appropriate evaluation, diagnosis, and management. |
|  | 8 | If I have drusen, does that mean I have age-related macular degeneration? | Drusen are small yellow or white deposits that accumulate under the retina, and they are a hallmark characteristic of age-related macular degeneration (AMD). However, the presence of drusen alone does not necessarily mean that you have AMD. There are two main types of AMD: dry AMD and wet AMD. Dry AMD is characterized by the presence of drusen and gradual breakdown or thinning of the macula, which is the central part of the retina responsible for sharp, central vision. Wet AMD involves the growth of abnormal blood vessels beneath the retina, leading to leakage and potential damage to the macula. While drusen are a common early sign of AMD, their presence does not guarantee the development of the disease. Many people, especially older individuals, may have some drusen without experiencing significant vision problems. Regular eye exams are essential for monitoring the progression of drusen and identifying any signs of AMD. If you have drusen or are concerned about your eye health, it's crucial to consult with an eye care professional, such as an ophthalmologist or optometrist. They can assess your condition, perform necessary tests, and provide appropriate guidance on how to manage and monitor your eye health. Early detection and management are crucial in addressing age-related macular degeneration and preserving vision. |
| Causes and risk factors | 9 | What causes age-related macular degeneration? | Age-related macular degeneration (AMD) is a progressive eye condition that primarily affects older individuals and can result in the loss of central vision. The exact cause of AMD is not fully understood, but it is believed to involve a combination of genetic, environmental, and lifestyle factors. Here are some key factors associated with the development of age-related macular degeneration: 1. **Age:** As the name suggests, AMD is primarily associated with aging. The risk of developing AMD increases with advancing age, and it is more commonly observed in individuals over the age of 50. 2. **Genetics:** There is evidence to suggest that genetics play a role in the development of AMD. Individuals with a family history of the condition may be at a higher risk. Certain genetic variations have been identified as potential risk factors for AMD.  3. **Smoking:** Smoking is a significant modifiable risk factor for AMD. Smokers have been shown to be at a higher risk of developing the condition compared to non-smokers. The harmful substances in tobacco smoke can contribute to oxidative stress and damage to the retina.  4. **Nutrition:** Diet can influence the risk of AMD. A diet rich in antioxidants, vitamins (especially vitamins C and E), and minerals (such as zinc and copper) may help protect against AMD. Conversely, a diet low in these nutrients may be associated with an increased risk.  5. **Cardiovascular Health:** Conditions that affect the cardiovascular system, such as hypertension and atherosclerosis, may increase the risk of AMD. Maintaining overall cardiovascular health may have a positive impact on eye health.  6. **Sunlight Exposure:** Prolonged exposure to ultraviolet (UV) light may contribute to the development and progression of AMD. Wearing sunglasses that block UV rays and protecting the eyes from excessive sunlight may be beneficial.  It's important to note that while these factors are associated with AMD, the exact interplay and mechanisms are still an active area of research. Additionally, not everyone with these risk factors will develop AMD, and some individuals without apparent risk factors may still develop the condition. Regular eye examinations are crucial for early detection and management of age-related macular degeneration. If you have concerns about your eye health, it's recommended to consult with an eye care professional for personalized advice and guidance. |
|  | 10 | What are the risk factors for age-related macular degeneration? | Age-related macular degeneration (AMD) is a progressive eye condition that primarily affects older adults and can lead to vision loss. Several risk factors are associated with the development and progression of AMD. These include:  1. **Age:** The risk of AMD increases with age, especially in individuals over 50.  2. **Family History:** If you have a family history of AMD, you may be at a higher risk. Genetic factors can contribute to the development of the condition.  3. **Race:** AMD is more common among Caucasians compared to other ethnic groups.  4. **Gender:** Women may be at a slightly higher risk than men.  5. **Smoking:** Smoking is a significant risk factor for AMD. It can double or even triple the risk of developing the condition.  6. **Nutrition:** A diet low in antioxidants, vitamins, and minerals may contribute to the development of AMD. A diet rich in green, leafy vegetables, and fish may be protective.  7. **Sunlight Exposure:** Prolonged exposure to sunlight, especially without protection, may increase the risk of AMD.  8. **Cardiovascular Disease:** Conditions such as hypertension and atherosclerosis may increase the risk of AMD.  9. **Obesity:** Being overweight or obese is associated with a higher risk of AMD.  10. **High Blood Pressure:** Hypertension is a risk factor for AMD.  11. **High Cholesterol:** Elevated levels of cholesterol may contribute to the development of AMD.  It's important to note that while these factors are associated with an increased risk, they don't guarantee the development of AMD. Conversely, some individuals may develop AMD without having any of these risk factors. Regular eye exams are crucial, especially for individuals with one or more risk factors, to detect AMD early and manage it effectively. If you have concerns about AMD or its risk factors, it's advisable to consult with an eye care professional for personalized advice and guidance. |
|  | 11 | Is there any way to prevent age-related macular degeneration? | While it's not possible to completely prevent age-related macular degeneration (AMD), there are several lifestyle and dietary measures that may help reduce the risk or slow down the progression of the disease. Keep in mind that these suggestions are based on general recommendations, and it's essential to consult with a healthcare professional for personalized advice. Here are some tips:  1. **Eat a Healthy Diet:**  - Include a variety of fruits and vegetables in your diet, especially those rich in antioxidants such as lutein and zeaxanthin. These are found in leafy greens like kale and spinach.  - Consume omega-3 fatty acids, which are found in fish (such as salmon and mackerel), flaxseeds, and walnuts.  2. **Maintain a Healthy Weight:**  - Obesity has been linked to an increased risk of AMD. Adopting a healthy diet and engaging in regular physical activity can help maintain a healthy weight.  3. **Exercise Regularly:**  - Regular physical activity may contribute to overall eye health. Aim for at least 150 minutes of moderate-intensity exercise per week.  4. **Protect Your Eyes from UV Light:**  - Wear sunglasses that block harmful UV rays to protect your eyes from potential damage caused by sunlight.  5. **Don't Smoke:**  - Smoking is a significant risk factor for AMD. Quitting smoking can reduce your risk and improve overall eye health.  6. **Manage Blood Pressure and Cholesterol:**  - High blood pressure and high cholesterol levels may contribute to AMD. Regular monitoring and management of these factors are essential.  7. **Control Blood Sugar:**  - For individuals with diabetes, proper management of blood sugar levels is crucial, as uncontrolled diabetes can contribute to vision problems, including AMD.  8. **Have Regular Eye Exams:**  - Regular eye check-ups can help detect AMD or other eye conditions in their early stages, allowing for timely intervention.  9. **Consider Eye Supplements:**  - Some studies suggest that certain nutritional supplements, such as vitamins and minerals (e.g., vitamin C, vitamin E, zinc, and copper), may help reduce the risk of AMD in certain individuals. However, it's essential to consult with an eye care professional before taking any supplements, as they may not be suitable for everyone.  Remember that while these measures may reduce the risk or slow the progression of AMD, they do not guarantee complete prevention. Genetics also play a role in the development of AMD, so it's essential to be aware of your family history and discuss any concerns with your healthcare provider. |
|  | 12 | What can I do to reduce my risk of age-related macular degeneration progression? | Age-related macular degeneration (AMD) is a progressive eye condition that primarily affects older adults and can lead to vision loss. While there is no guaranteed way to prevent AMD, certain lifestyle and dietary changes may help reduce the risk of progression. Here are some tips:  1. **Eat a Healthy Diet:**  - Consume a diet rich in fruits and vegetables, especially those high in antioxidants like vitamins C and E, zinc, and lutein. Leafy greens, berries, and carrots are good choices.  - Include fish high in omega-3 fatty acids, such as salmon and trout, which may have protective effects on the eyes.  2. **Maintain a Healthy Weight:**  - Obesity is a risk factor for AMD. Adopting a healthy lifestyle that includes regular exercise and a balanced diet can help maintain a healthy weight and reduce the risk of AMD.  3. **Quit Smoking:**  - Smoking is a significant risk factor for AMD progression. If you smoke, quitting can help reduce your risk and improve overall eye health.  4. **Protect Your Eyes from UV Light:**  - Wear sunglasses that block ultraviolet (UV) radiation to protect your eyes from harmful sun exposure. Prolonged exposure to UV light may contribute to the development and progression of AMD.  5. **Manage Cardiovascular Health:**  - Conditions like high blood pressure and high cholesterol may increase the risk of AMD. Regular exercise, a heart-healthy diet, and managing cardiovascular risk factors can contribute to overall eye health.  6. **Control Blood Sugar Levels:**  - For individuals with diabetes, managing blood sugar levels is crucial. Uncontrolled diabetes can contribute to the development and progression of AMD.  7. **Take Eye Health Supplements:**  - Some studies suggest that specific supplements, such as those containing vitamins C and E, zinc, copper, and antioxidants like lutein and zeaxanthin, may help slow the progression of AMD. Consult with your eye care professional before starting any supplements.  8. **Get Regular Eye Check-ups:**  - Regular eye examinations are crucial for detecting AMD early. Early intervention can help manage the condition and slow down its progression.  9. **Limit Alcohol Consumption:**  - Excessive alcohol consumption may contribute to AMD progression. If you drink alcohol, do so in moderation.  10. **Follow Treatment Plans:**  - If you have been diagnosed with AMD, follow your eye care professional's recommendations and treatment plans. Regular monitoring and prompt intervention can help manage the condition.  It's essential to consult with your eye care professional for personalized advice and recommendations based on your specific situation. They can provide guidance tailored to your eye health needs. |
|  | 13 | Are my family members at risk for age-related macular degeneration if I have it? | Age-related macular degeneration (AMD) does have a genetic component, so if you have been diagnosed with AMD, there may be a slightly increased risk for your family members. However, it's important to note that AMD is influenced by a combination of genetic and environmental factors.  Several genes have been associated with an increased risk of AMD, and having a family history of the condition can contribute to your own risk. If multiple family members have been diagnosed with AMD, it may suggest a genetic predisposition.  However, lifestyle factors also play a significant role in the development and progression of AMD. Smoking, poor diet, and other environmental factors can contribute to the risk of AMD.  If you have concerns about the risk of AMD in your family, it's recommended to discuss these concerns with your eye care professional. Regular eye exams are crucial for early detection and management of AMD, and your eye doctor can provide personalized advice based on your family history, overall health, and lifestyle factors. Additionally, they may recommend lifestyle modifications to reduce the risk of AMD, such as maintaining a healthy diet rich in antioxidants, protecting your eyes from UV light, and not smoking. |
|  | 14 | What causes age-related macular degeneration to get worse? | Age-related macular degeneration (AMD) is a progressive eye condition that primarily affects individuals over the age of 50. The exact cause of AMD is not fully understood, but a combination of genetic, environmental, and lifestyle factors is believed to contribute to its development and progression.  Factors that may contribute to the worsening of age-related macular degeneration include:  1. **Genetics:** Family history and genetics play a significant role in the development of AMD. If you have close relatives with AMD, you may be at a higher risk of developing the condition and experiencing its progression.  2. **Age:** As the name suggests, AMD is age-related, and the risk increases with advancing age. The degeneration of the macula, which is responsible for central vision, tends to progress over time.  3. **Smoking:** Smoking is a major risk factor for AMD and is associated with an increased likelihood of progression. If you smoke, quitting may help slow down the deterioration.  4. **Poor Diet:** A diet lacking in certain nutrients, such as antioxidants, vitamins, and minerals, may contribute to the progression of AMD. Diets rich in green leafy vegetables, fish, and other sources of antioxidants are thought to be beneficial.  5. **Cardiovascular Health:** Conditions that affect cardiovascular health, such as hypertension and high cholesterol, may contribute to the progression of AMD. Managing these conditions through a healthy lifestyle and medical interventions may help slow down AMD.  6. **Sun Exposure:** Prolonged exposure to ultraviolet (UV) light may contribute to AMD progression. Wearing sunglasses that block harmful UV rays when outdoors may be beneficial.  7. **Obesity:** Being overweight or obese is associated with an increased risk of AMD progression. Maintaining a healthy weight through diet and exercise may help manage the condition.  8. **Inflammation:** Chronic inflammation in the body may contribute to the progression of AMD. Anti-inflammatory measures, such as a healthy diet and regular exercise, may have a positive impact.  It's important for individuals with AMD to work closely with their eye care professionals to monitor their condition and discuss strategies for managing and slowing down its progression. Regular eye exams, lifestyle modifications, and adherence to treatment plans can help address specific risk factors and maintain eye health. |
| Symptoms and detection | 15 | What is an Amsler grid and how do I use it for age-related macular degeneration? | An Amsler grid is a tool used to monitor and detect changes in central vision, particularly for individuals with age-related macular degeneration (AMD). AMD is a degenerative eye disease that primarily affects the macula, the central part of the retina responsible for sharp, central vision.  To use an Amsler grid for AMD:  1. **Prepare the Amsler grid:**  - The grid is typically a square with a central dot. The grid lines may appear as straight lines, forming a checkerboard pattern.  - Ensure good lighting conditions when using the grid.  2. **Wear reading glasses if needed:**  - If you use reading glasses, wear them while using the Amsler grid.  3. **Position the grid at eye level:**  - Hold the grid at a comfortable reading distance, usually about 12-14 inches (30-35 cm) away from your eyes.  4. **Cover one eye:**  - To assess each eye independently, cover one eye with your hand or an eye patch.  5. **Focus on the central dot:**  - Keep your gaze fixed on the central dot in the middle of the grid.  6. **Observe grid lines:**  - While focusing on the central dot, pay attention to the grid lines and any areas where the lines appear distorted, wavy, or missing.  7. **Note abnormalities:**  - If you notice any changes, such as blurry, distorted, or missing areas in the grid, make a note of their location on the grid.  8. **Repeat with the other eye:**  - Repeat the process, covering the other eye and observing any changes.  9. **Regular monitoring:**  - Perform this self-check regularly, such as daily or weekly, depending on your eye care professional's recommendations.  The Amsler grid is a valuable tool for detecting changes in central vision and alerting individuals to potential issues with AMD. If you notice any changes or abnormalities, it's crucial to consult with an eye care professional promptly for further evaluation and management. Early detection and intervention are essential in managing AMD and preserving vision. |
|  | 16 | Can age-related macular degeneration lead to blindness? | Yes, age-related macular degeneration (AMD) can potentially lead to blindness, particularly in its advanced stages. AMD is a progressive eye condition that primarily affects the macula, the central part of the retina responsible for sharp, central vision. There are two main types of AMD: dry AMD and wet AMD.  1. Dry AMD: This is the more common form, and it typically progresses slowly. In dry AMD, small yellow deposits known as drusen accumulate in the macula, leading to a gradual deterioration of vision. While dry AMD can cause significant vision loss, it often progresses more slowly than wet AMD.  2. Wet AMD: Although less common than dry AMD, wet AMD is more severe and can progress rapidly. In wet AMD, abnormal blood vessels grow beneath the retina and leak blood and fluid, leading to more rapid and severe vision loss. If left untreated, wet AMD can result in significant damage to the macula and central vision, potentially leading to legal blindness.  It's important for individuals with AMD to undergo regular eye examinations to monitor the progression of the condition. While there is no cure for AMD, certain treatments, especially for wet AMD, such as anti-VEGF (vascular endothelial growth factor) injections, may help slow down the progression of the disease and preserve vision. Early detection and management are crucial in minimizing the risk of severe vision impairment or blindness associated with age-related macular degeneration. |
|  | 17 | What are the symptoms of age-related macular degeneration? | Age-related macular degeneration (AMD) is a progressive eye condition that primarily affects older adults and can lead to vision loss. There are two main types of AMD: dry AMD and wet AMD. The symptoms may vary depending on the type, but both can cause significant visual impairment. Here are the common symptoms associated with each type:  1. **Dry AMD:**  - Gradual central vision loss: Dry AMD typically progresses slowly, causing a gradual loss of central vision over time.  - Blurred or fuzzy vision: Patients may experience difficulty seeing fine details or sharp lines.  - Difficulty recognizing faces: Due to central vision impairment, recognizing faces may become challenging.  - Distorted vision: Straight lines may appear wavy or distorted.  2. **Wet AMD:**  - Sudden and severe central vision loss: Wet AMD can cause a rapid decline in central vision, often more pronounced than in dry AMD.  - Distorted vision: Similar to dry AMD, straight lines may appear wavy or distorted.  - Increased size or number of blind spots: Patients may notice an enlargement of blind spots in their visual field.  - Visual hallucinations (rare): In some cases, individuals with wet AMD may experience hallucinations, known as Charles Bonnet syndrome.  It's crucial to note that AMD primarily affects central vision, leaving peripheral vision relatively intact. Early detection and management are crucial in preventing further vision loss. Regular eye exams are essential, especially for individuals over the age of 50 or those with a family history of AMD. If you experience any changes in your vision, it's important to consult with an eye care professional promptly. |
|  | 18 | How is age-related macular degeneration diagnosed? | Age-related macular degeneration (AMD) is typically diagnosed through a combination of medical history, eye examinations, and imaging tests. Here are the key components of the diagnostic process for AMD:  1. **Comprehensive Eye Exam:**  - An eye care professional will conduct a thorough eye examination, which may include checking visual acuity, dilating the pupils to examine the retina, and assessing the overall health of the eyes.  2. **Dilated Fundus Examination:**  - During a dilated fundus examination, the eye care professional uses special eye drops to dilate the pupils, allowing for a better view of the back of the eye, including the macula and retina.  3. **Amsler Grid Test:**  - The Amsler grid is a tool that patients may use at home to self-monitor their vision. It consists of a grid with straight lines, and patients with AMD may notice distortions or missing areas when looking at the grid.  4. **Fluorescein Angiography:**  - This imaging test involves injecting a special dye (fluorescein) into a vein, usually in the arm. As the dye circulates through the blood vessels in the eye, a series of photographs are taken to highlight any abnormalities, such as leaking blood vessels.  5. **Optical Coherence Tomography (OCT):**  - OCT is a non-invasive imaging technique that provides detailed cross-sectional images of the retina. It is particularly useful for detecting and monitoring changes in the macula, such as the presence of drusen (yellow deposits under the retina) and fluid accumulation.  6. **Fundus Photography:**  - High-resolution photographs of the back of the eye can be taken to document the appearance of the macula and retina. These images can be compared over time to track any changes.  7. **Visual Acuity Testing:**  - Standard eye charts are used to measure visual acuity at different distances. Changes in vision can be indicative of AMD progression.  8. **Genetic Testing:**  - In some cases, genetic testing may be recommended, especially if there is a family history of AMD. Certain genetic factors can increase the risk of developing AMD.  It's important to note that early detection and diagnosis of AMD are crucial for managing the condition effectively. If someone notices changes in their vision or is at risk due to age or family history, they should promptly consult an eye care professional for a comprehensive eye examination. Regular eye check-ups are also essential for individuals over the age of 50, even if they do not have noticeable vision problems. |
|  | 19 | How often do I need to get checked for age-related macular degeneration? | The frequency of eye exams to check for age-related macular degeneration (AMD) depends on your age, risk factors, and any existing eye conditions. In general, regular eye exams are important for maintaining overall eye health, and they become especially crucial as you age.  The American Academy of Ophthalmology (AAO) recommends the following guidelines for eye exams:  1. **Age 40-54:** Every 2-4 years 2. **Age 55-64:** Every 1-3 years 3. **Age 65 and older:** Every 1-2 years  However, if you have risk factors for AMD, such as a family history of the disease, smoking, or certain genetic factors, your eye doctor may recommend more frequent exams. Individuals with existing eye conditions or diseases may also need more frequent monitoring.  It's important to note that these are general guidelines, and your eye care professional may tailor the frequency of your eye exams based on your individual health and risk factors. Always follow the recommendations of your eye care provider for personalized guidance on how often you should get checked for AMD. |
|  | 20 | How quickly does age-related macular degeneration progress? | The progression of age-related macular degeneration (AMD) can vary significantly from person to person. AMD is a chronic and progressive eye condition that primarily affects the macula, the central part of the retina responsible for sharp, central vision. There are two main types of AMD: dry AMD and wet AMD.  1. **Dry AMD:** This is the more common form of AMD, and it tends to progress slowly over many years. In some cases, it may not progress much at all, while in others, it can lead to significant vision loss over time.  2. **Wet AMD:** This form of AMD is characterized by the growth of abnormal blood vessels beneath the macula. Wet AMD can cause more rapid and severe vision loss than dry AMD. It can progress quickly and may lead to a sudden and significant decline in central vision.  Several factors can influence the rate of progression, including genetics, lifestyle choices, and overall health. Risk factors for AMD include age, family history, smoking, and certain genetic factors. Regular eye exams are crucial for detecting AMD early, allowing for better management and potential intervention.  It's important for individuals at risk of AMD or those diagnosed with early stages to have regular check-ups with an eye care professional. Early detection and appropriate management strategies, such as lifestyle changes and sometimes medical treatments, can help slow down the progression and reduce the risk of severe vision loss. |
|  | 21 | Can I still drive if I have age-related macular degeneration? | Age-related macular degeneration (AMD) can affect central vision and may impact the ability to drive safely. The severity of AMD varies among individuals, and it's essential to assess your specific situation with the guidance of an eye care professional.  If you have AMD and are concerned about your ability to drive, consider the following steps:  1. **Consult an Eye Care Professional:**  - Schedule regular eye exams with an ophthalmologist or optometrist to monitor the progression of AMD.  - Discuss your concerns about driving with your eye care professional, who can evaluate your visual function and provide guidance based on your specific case.  2. **Visual Acuity and Field of Vision:**  - AMD can affect central vision, which is crucial for tasks like reading road signs and recognizing hazards.  - Your eye care professional may assess your visual acuity and field of vision to determine if it meets the legal requirements for driving in your location.  3. **Adaptations and Assistive Devices:**  - Some individuals with AMD may benefit from visual aids or adaptive devices that can enhance their ability to see and navigate the road.  - Discuss with your eye care professional if such devices could be helpful in your case.  4. **Legal Requirements:**  - Be aware of the legal requirements for driving in your area. Different regions have specific vision standards for obtaining and maintaining a driver's license.  - If your vision falls below the legal requirements, it may impact your ability to drive.  5. **Driver's License Renewal:**  - Regularly check the renewal requirements for your driver's license. Some jurisdictions may have specific vision tests during the renewal process.  6. **Consider Alternative Transportation:**  - If it's determined that your vision no longer meets the requirements for safe driving, consider alternative transportation options such as public transit, rideshare services, or assistance from friends and family.  Ultimately, the decision about whether you can still drive with AMD should be made in consultation with your eye care professional and in accordance with local regulations. It's important to prioritize safety on the road for yourself and others. |
|  | 22 | Can artificial intelligence diagnose age-related macular degeneration? | Artificial intelligence (AI) has shown promise in assisting with the diagnosis and management of age-related macular degeneration (AMD). AMD is a common eye condition that affects the central part of the retina, known as the macula, leading to vision loss over time. AI can be employed in various ways to aid in the detection and monitoring of AMD:  1. **Image Analysis:** AI algorithms can analyze retinal images, such as fundus photographs or optical coherence tomography (OCT) scans, to identify signs of AMD. These algorithms can detect specific features associated with AMD, such as drusen (yellow deposits beneath the retina), pigmentary changes, or fluid accumulation.  2. **Early Detection:** AI can potentially assist in the early detection of AMD by identifying subtle changes in retinal images that may not be easily noticeable to the human eye. Early detection is crucial for timely intervention and better management of the condition.  3. **Monitoring Progression:** AI systems can be trained to monitor changes in the retina over time, helping healthcare professionals track the progression of AMD. This continuous monitoring is important for adapting treatment plans and interventions as needed.  4. **Risk Prediction:** AI models can also be developed to assess the risk of developing AMD based on various factors such as age, genetics, lifestyle, and medical history. This predictive capability could enable personalized preventive measures for individuals at higher risk.  Several research studies and commercial projects are exploring the use of AI in AMD diagnosis and management. However, it's important to note that AI systems are not a replacement for healthcare professionals. They are intended to assist and augment the capabilities of medical practitioners by providing efficient and accurate analysis of medical images.  Before AI-based diagnostic tools for AMD are widely adopted, thorough validation and regulatory approval processes are typically required to ensure their reliability, safety, and effectiveness in real-world clinical settings. Healthcare professionals should always be involved in the final decision-making and interpretation of results. |
|  | 23 | Do I need the dye injection test to diagnose age-related macular degeneration? | The dye injection test you are likely referring to is called fluorescein angiography. Fluorescein angiography is a diagnostic test that involves injecting a special dye, fluorescein, into a vein in your arm. The dye travels through the blood vessels in the retina, allowing the ophthalmologist to capture detailed images of the blood flow in the back of the eye.  Fluorescein angiography is commonly used to diagnose and monitor various eye conditions, including age-related macular degeneration (AMD). AMD is a degenerative disease that affects the macula, the central part of the retina responsible for sharp, central vision.  While fluorescein angiography can provide valuable information about blood flow and identify abnormal blood vessel growth (neovascularization) in the retina, it is not always necessary for the diagnosis of AMD. In many cases, AMD can be diagnosed through a comprehensive eye examination that includes a dilated eye exam, visual acuity testing, and optical coherence tomography (OCT).  Optical coherence tomography is a non-invasive imaging technique that provides cross-sectional images of the retina, helping to assess the thickness and structure of the macula. It is often used as a part of the diagnostic process for AMD.  The decision to perform fluorescein angiography depends on the specific details of your case and the judgment of your eye care professional. They will consider factors such as the severity of your symptoms, the type of AMD suspected, and other clinical findings.  If you have concerns about your vision or are at risk for AMD, it is important to schedule a comprehensive eye examination with an eye care professional. They can determine the most appropriate diagnostic tests based on your individual circumstances. |
| Treatment and follow up | 24 | How is dry age-related macular degeneration treated? | As of my last knowledge update in January 2022, dry age-related macular degeneration (AMD) does not have a cure, and treatment focuses on managing symptoms and slowing the progression of the disease. It's important to note that medical advancements may have occurred since then, so it's advisable to consult with a healthcare professional for the latest information.  As of my last update, here are some common approaches to managing dry AMD:  1. **Lifestyle Modifications:**  - **Nutrition:** Some studies have suggested that certain vitamins and minerals, such as vitamin C, vitamin E, zinc, copper, and antioxidants, may help slow the progression of AMD. Eye supplements containing these nutrients may be recommended.  - **Diet:** A diet rich in leafy green vegetables, fish, and other foods high in omega-3 fatty acids may be beneficial.  2. **Low Vision Aids:**  - Devices such as magnifying glasses, telescopic lenses, or electronic magnifiers can help individuals with AMD make the most of their remaining vision.  3. **Supportive Therapies:**  - Occupational therapy can provide strategies to cope with vision loss and maintain independence in daily activities.  4. **Anti-VEGF Injections (Wet AMD Only):**  - For wet AMD, which involves abnormal blood vessel growth, anti-VEGF (vascular endothelial growth factor) medications may be injected into the eye to inhibit the growth of new blood vessels and reduce leakage.  It's crucial to undergo regular eye exams to monitor the progression of AMD and adjust the treatment plan accordingly. If you or someone you know is experiencing symptoms of AMD, it is essential to consult with an eye care professional for a proper diagnosis and personalized treatment plan. Keep in mind that ongoing research and advancements in medical science may bring about new treatments or approaches for managing dry AMD. |
|  | 25 | How is wet-age-related macular degeneration treated? | Wet age-related macular degeneration (wet AMD) is a chronic eye disorder that can cause central vision loss. It is characterized by the growth of abnormal blood vessels under the macula, the central part of the retina. These vessels can leak fluid and blood, leading to damage of the macula and loss of central vision. The primary goal of treatment for wet AMD is to stop the growth of these abnormal blood vessels and prevent further damage to the macula. Here are some common treatment options:  1. **Anti-VEGF Injections:** Anti-VEGF (vascular endothelial growth factor) drugs are the most common and effective treatment for wet AMD. These drugs, including ranibizumab, bevacizumab, and aflibercept, are injected directly into the eye. They work by inhibiting the growth of abnormal blood vessels, reducing leakage, and preserving vision. Multiple injections are usually needed over time, and the frequency of injections varies based on the individual response to treatment.  2. **Photodynamic Therapy (PDT):** PDT involves injecting a light-sensitive drug into the bloodstream, which is then activated by laser light aimed at the abnormal blood vessels in the eye. This treatment is less common than anti-VEGF therapy and may be used in certain cases.  3. **Laser Therapy:** Laser treatment can be used to destroy abnormal blood vessels in some cases, but it is less commonly employed due to the risk of damaging surrounding healthy tissue.  4. **Implantable Devices:** In some cases, implantable devices may be used to deliver sustained release of anti-VEGF drugs into the eye, reducing the need for frequent injections.  It's important to note that while these treatments can help slow or halt the progression of wet AMD, they may not fully restore lost vision. Early detection and prompt treatment are crucial for the best possible outcomes. Regular eye exams are recommended, especially for individuals at higher risk of AMD, such as those with a family history of the disease or individuals over the age of 50. If you or someone you know is experiencing symptoms of AMD, such as distorted or blurred central vision, it's important to consult with an eye care professional for a thorough examination and appropriate management. |
|  | 26 | Is age-related macular degeneration curable? | As of my last knowledge update in January 2022, age-related macular degeneration (AMD) is generally considered incurable. AMD is a chronic eye condition that primarily affects older adults and can lead to vision loss over time. There are two main types of AMD: dry AMD and wet AMD.  1. **Dry AMD:** This is the more common form, and there is currently no cure for it. However, there are some treatments aimed at slowing its progression, and certain nutritional supplements may be recommended.  2. **Wet AMD:** This form involves the growth of abnormal blood vessels beneath the macula, leading to more severe vision loss. There are some treatments for wet AMD, including anti-VEGF drugs, which can help slow down or halt the growth of these blood vessels. While these treatments can be effective in managing the condition, they are not considered a cure.  Research and medical advancements are ongoing, and new treatments may have been developed since my last update. It's essential to consult with an eye care professional for the latest information on AMD and available treatments. Keep in mind that even with treatment, the goal may be to slow the progression of the disease and manage symptoms rather than achieve a complete cure. |
|  | 27 | Do all types of age-related macular degeneration need injections? | Age-related macular degeneration (AMD) is a progressive eye condition that can affect the central part of the retina, called the macula, leading to loss of central vision. There are two main types of AMD: dry AMD and wet AMD.  1. **Dry AMD:** This form is more common and generally progresses slowly. Currently, there is no specific treatment for dry AMD, and it does not typically involve injections. However, certain nutritional supplements, such as vitamins and minerals (e.g., AREDS formula), may be recommended to slow the progression of dry AMD.  2. **Wet AMD:** This form is less common but tends to progress more rapidly and is associated with abnormal blood vessel growth under the retina. In wet AMD, injections of anti-VEGF drugs into the eye are a common and effective treatment. Anti-VEGF drugs help to block the growth of abnormal blood vessels and reduce leakage, slowing down vision loss and sometimes even improving vision.  The decision to use injections as a treatment for wet AMD is made based on the specific characteristics of the disease and the assessment by an eye care professional. It's important to note that the injection treatment doesn't cure AMD, but it can help manage the symptoms and slow down the progression of the disease.  It's crucial for individuals with AMD to have regular eye exams and work closely with their eye care team to determine the most appropriate treatment plan for their specific condition. Treatment approaches may evolve over time as new research and therapies are developed. |
|  | 28 | Can laser eye surgery treat age-related macular degeneration? | As of my last knowledge update in January 2022, laser eye surgery, such as LASIK (Laser-Assisted In Situ Keratomileusis), is not a treatment for age-related macular degeneration (AMD). AMD is a degenerative eye condition that affects the macula, the central part of the retina responsible for sharp, central vision. It is primarily associated with aging.  Laser eye surgery like LASIK is designed to correct refractive errors such as nearsightedness, farsightedness, and astigmatism by reshaping the cornea. It does not address the underlying issues related to AMD, which involve damage to the macula and its supporting structures.  The treatment options for AMD typically include medications, injections, and in some cases, laser therapy. Anti-VEGF drugs, which are injected into the eye, have been commonly used to slow the progression of certain types of AMD.  It's crucial to consult with an eye care professional or a retinal specialist for an accurate diagnosis and appropriate treatment recommendations tailored to the specific type and stage of macular degeneration. Keep in mind that advancements in medical treatments may occur, so it's always a good idea to check for updates or new developments in the field of ophthalmology. |
|  | 29 | How many treatments do I need for wet age-related macular degeneration? | The treatment plan for wet age-related macular degeneration (AMD) can vary based on individual circumstances, and it is crucial to consult with your eye care professional for personalized advice. Typically, the most common treatments for wet AMD involve anti-VEGF (vascular endothelial growth factor) medications, which are injected into the eye to help slow down or prevent the growth of abnormal blood vessels.  The frequency of anti-VEGF injections can vary, and your eye care specialist will determine the appropriate treatment schedule based on the severity of your condition, response to treatment, and other factors. Initially, injections may be more frequent, and as the condition stabilizes, the frequency might be reduced. Some individuals may require ongoing treatment, while others may experience longer intervals between injections.  It's important to attend regular follow-up appointments with your eye care professional to monitor the progression of the disease and adjust the treatment plan as needed. Keep in mind that advancements in AMD treatment may occur over time, so your eye care specialist will consider the most current and effective options for managing wet AMD. |
|  | 30 | What are the benefits and how successful is the treatment for wet age-related macular degeneration? | As of my last knowledge update in January 2022, wet age-related macular degeneration (wet AMD) is a chronic eye condition that can cause severe vision loss if left untreated. The primary treatment for wet AMD involves the use of anti-vascular endothelial growth factor (anti-VEGF) medications. These drugs, such as ranibizumab (Lucentis), aflibercept (Eylea), and bevacizumab (Avastin), are injected into the eye to inhibit the growth of abnormal blood vessels and reduce leakage, which are characteristic features of wet AMD.  Benefits of Anti-VEGF Treatment for Wet AMD:  1. **Vision Improvement:** Anti-VEGF injections can often lead to improvements in vision for individuals with wet AMD. In some cases, patients may experience stabilization of their vision, while others may see a significant improvement.  2. **Prevention of Further Vision Loss:** The primary goal of treatment is to prevent further damage to the macula and preserve existing vision.  3. **Quality of Life:** Maintaining or improving vision can positively impact an individual's overall quality of life, allowing them to continue with daily activities and maintain independence.  Success and Effectiveness:  The effectiveness of anti-VEGF treatment can vary among individuals, and not everyone responds in the same way. Some patients experience significant improvement, while others may have more modest gains. It's important to note that the success of treatment often depends on factors such as the stage of AMD, the extent of damage to the macula, and the patient's overall health.  Continuous Treatment and Monitoring:  Anti-VEGF treatment is usually ongoing, requiring regular injections and monitoring by an eye care professional. The frequency of injections may vary depending on the specific drug and the individual's response to treatment.  While anti-VEGF injections have shown significant success in managing wet AMD, it's essential for individuals with this condition to work closely with their eye care team to determine the most appropriate treatment plan for their specific case.  It's advisable to consult with an eye care professional for the latest information and updates on wet AMD treatments, as new developments and research may have occurred since my last knowledge update in January 2022. |
|  | 31 | What are the risks and side effects associated with age-related macular degeneration treatment? | Age-related macular degeneration (AMD) is a progressive eye condition that can lead to severe vision loss, particularly in older adults. There are different treatment options available for AMD, and each comes with its own set of risks and side effects. It's important to note that the specific risks and side effects can vary depending on the type of AMD and the chosen treatment. Here are some common treatments and their associated risks:  1. **Anti-VEGF Injections:**  - **Risks:** The most common treatment for neovascular or wet AMD involves injecting anti-vascular endothelial growth factor (anti-VEGF) medications (such as ranibizumab, aflibercept, and bevacizumab) into the eye. While generally effective, there are risks, such as infection, bleeding, and increased eye pressure.  - **Side Effects:** Common side effects may include temporary discomfort, redness, or swelling at the injection site. Some patients may experience floaters or blurry vision, but these effects usually resolve on their own.  2. **Photodynamic Therapy (PDT):**  - **Risks:** PDT involves using a light-activated drug to damage abnormal blood vessels in the eye. Risks may include sensitivity to light, skin reactions, and damage to healthy blood vessels.  - **Side Effects:** Side effects can include temporary visual disturbances, pain or discomfort during the procedure, and, rarely, scarring.  3. **Laser Therapy:**  - **Risks:** Laser therapy is used to seal leaking blood vessels. Risks include scarring and potential damage to the surrounding healthy tissue.  - **Side Effects:** Side effects may include temporary discomfort, blurry vision, and sensitivity to light.  4. **Implantable Telescope:**  - **Risks:** For advanced AMD, an implantable miniature telescope can be implanted into the eye to improve central vision. Risks include infection, bleeding, and potential damage to the eye structures.  - **Side Effects:** Common side effects may include glare and halos, and patients may need time to adapt to the changes in vision.  It's crucial to discuss the potential risks and side effects with your ophthalmologist before undergoing any AMD treatment. The decision to proceed with a particular treatment depends on factors such as the type and stage of AMD, overall health, and individual preferences. Regular monitoring and follow-up appointments are essential to assess the effectiveness of the chosen treatment and address any concerns or complications that may arise. |
|  | 32 | What lifestyle factors can help manage my age-related macular degeneration? | Age-related macular degeneration (AMD) is a progressive eye condition that can result in central vision loss. While there is no cure for AMD, certain lifestyle factors may help manage the condition and potentially slow its progression. It's important to note that these recommendations are general in nature, and individual cases may vary. Consult with your eye care professional for personalized advice. Here are some lifestyle factors that may be beneficial:  1. **Healthy Diet:**  - Include foods rich in antioxidants, vitamins, and minerals, such as leafy green vegetables, fruits, and fish. Omega-3 fatty acids found in fish like salmon and trout may be particularly helpful.  2. **Eye-Healthy Nutrients:**  - Consider supplements with specific nutrients that support eye health, such as vitamins C and E, zinc, copper, and lutein/zeaxanthin. However, it's crucial to consult your eye care professional before starting any supplements, as they can interact with medications or have side effects.  3. **Maintain a Healthy Weight:**  - Obesity is associated with an increased risk of AMD progression. Adopting a healthy weight through a balanced diet and regular exercise may be beneficial.  4. **Regular Exercise:**  - Engaging in regular physical activity may promote overall health, including eye health. Exercise improves blood circulation, which can be beneficial for the eyes.  5. **Quit Smoking:**  - Smoking has been linked to an increased risk of developing AMD and can accelerate its progression. Quitting smoking may help slow down the disease.  6. **Protect Your Eyes from UV Light:**  - Wear sunglasses that block ultraviolet (UV) light to protect your eyes from harmful sun exposure. Prolonged exposure to UV rays may contribute to the development and progression of AMD.  7. **Manage Blood Pressure and Cholesterol:**  - High blood pressure and cholesterol levels may contribute to AMD progression. Managing these factors through a healthy diet, exercise, and medication if necessary can be beneficial.  8. **Regular Eye Exams:**  - Schedule regular eye exams with your eye care professional. Early detection and timely treatment of AMD-related changes may help manage the condition more effectively.  9. **Adequate Sleep:**  - Ensure you get enough quality sleep, as it is crucial for overall health and well-being, including eye health.  10. **Manage Stress:**  - Chronic stress may impact overall health, including eye health. Engage in stress-reducing activities such as meditation, yoga, or hobbies you enjoy.  It's essential to consult with your eye care professional for personalized advice based on your specific situation. They can provide guidance tailored to your individual needs and monitor any changes in your eye health over time. |
|  | 33 | Should I take AREDS supplementation for age-related macular degeneration? | I am not a doctor, but I can provide some general information. It's important to consult with a healthcare professional for personalized advice.  AREDS (Age-Related Eye Disease Study) is a clinical trial sponsored by the National Eye Institute in the United States. The study found that a specific combination of vitamins and minerals, known as the AREDS formulation, may help reduce the risk of progression of age-related macular degeneration (AMD) in certain individuals.  The original AREDS formulation included vitamin C, vitamin E, beta-carotene, zinc, and copper. However, beta-carotene was later replaced with lutein and zeaxanthin in the AREDS2 study for safety reasons, especially for current or former smokers, as beta-carotene has been associated with an increased risk of lung cancer in this population.  If you have AMD or are at risk of developing it, it's important to speak with your eye care professional before starting any supplementation. They can assess your specific situation and determine whether AREDS supplementation is appropriate for you. Factors such as the stage of AMD, overall health, and other medications you may be taking can influence this decision.  Additionally, a balanced diet rich in nutrients, including those found in fruits and vegetables, is generally beneficial for eye health. Lifestyle factors such as not smoking and protecting your eyes from harmful UV rays can also contribute to maintaining eye health.  Ultimately, only a healthcare professional can provide personalized advice based on your individual health status and needs. |
|  | 34 | What is the difference between AREDS and AREDS2 supplementation for age-related macular degeneration? | AREDS (Age-Related Eye Disease Study) and AREDS2 are two clinical trials sponsored by the National Eye Institute (NEI) in the United States, designed to investigate the effects of specific nutritional supplements on the progression of age-related macular degeneration (AMD), a leading cause of vision loss in older adults. Here are the key differences between AREDS and AREDS2 supplementation:  1. **Formulation:**  - **AREDS:** The original AREDS study, conducted between 2001 and 2013, evaluated a specific formulation containing high doses of antioxidants (vitamin C, vitamin E, and beta-carotene) and zinc (as zinc oxide). This combination was shown to reduce the risk of advanced AMD in individuals at high risk.    - **AREDS2:** Building on the AREDS findings, AREDS2, conducted from 2006 to 2012, aimed to refine the original formulation. It tested the addition of lutein and zeaxanthin (carotenoids found in green leafy vegetables) and examined the effects of reducing the zinc dose or eliminating beta-carotene due to concerns about its association with lung cancer in smokers.  2. **Components:**  - **AREDS:** The original formulation included vitamin C (500 mg), vitamin E (400 IU), beta-carotene (15 mg), zinc (as zinc oxide, 80 mg), and copper (as cupric oxide, 2 mg).   - **AREDS2:** This study tested four variations of the supplement. These variations included:  - The original AREDS formulation without beta-carotene.  - The original AREDS formulation with lower zinc (25 mg) and copper (0.5 mg) levels.  - The original AREDS formulation with lutein (10 mg) and zeaxanthin (2 mg) replacing beta-carotene.  - The original AREDS formulation with lower zinc, copper, and lutein/zeaxanthin instead of beta-carotene.  3. **Outcome:**  - **AREDS:** Showed that the original formulation reduced the risk of advanced AMD in high-risk individuals by about 25%.   - **AREDS2:** Found that the original AREDS formulation without beta-carotene and with lower zinc and copper levels, or with lutein/zeaxanthin instead of beta-carotene, were as effective as the original AREDS formulation in reducing the risk of advanced AMD. The lower zinc and copper levels in the modified formulations were found to be safer without compromising efficacy.  In summary, AREDS2 aimed to build upon the success of AREDS by refining the supplement formulation based on new research and addressing potential concerns. The modified formulations in AREDS2 were found to be effective in reducing the risk of advanced AMD while addressing safety considerations. Individuals with AMD should consult with their eye care professionals to determine the most appropriate supplementation based on their specific needs and health considerations. |
|  | 35 | Are there any eye drops available for treating age-related macular degeneration? | As of my last knowledge update in January 2022, there were no eye drops specifically approved for treating age-related macular degeneration (AMD). AMD is a complex eye condition that primarily affects the central part of the retina, known as the macula.  Treatment options for AMD typically include medications delivered through injections into the eye, such as anti-vascular endothelial growth factor (anti-VEGF) drugs. These injections help inhibit abnormal blood vessel growth and reduce leakage in the retina, which are characteristic features of AMD.  While research and development in the field of ophthalmology continue, it's essential to consult with an eye care professional or retinal specialist for the most up-to-date information on AMD treatments. New medications or therapies may have been developed since my last update, and your healthcare provider can provide guidance based on the latest advancements in the field. |
|  | 36 | What is the prognosis for people with age-related macular degeneration? | Age-related macular degeneration (AMD) is a progressive eye condition that affects the macula, a small area near the center of the retina. The macula is responsible for sharp, central vision, which is crucial for activities like reading and driving. There are two main types of AMD: dry AMD and wet AMD.  1. **Dry AMD (Non-neovascular AMD):** This is the more common form, accounting for about 80-90% of AMD cases. It progresses slowly, and vision loss is generally gradual. There is currently no cure for dry AMD, but certain lifestyle changes, dietary supplements (such as vitamins and minerals), and monitoring can help manage the condition and slow down its progression.  2. **Wet AMD (Neovascular AMD):** Although less common, wet AMD is more severe and can lead to rapid and significant vision loss. In wet AMD, abnormal blood vessels grow beneath the macula, causing leakage and damage to the retina. Treatments for wet AMD include anti-VEGF (vascular endothelial growth factor) injections and laser therapy, which can help slow down or even stabilize vision loss in some cases.  The prognosis for individuals with AMD varies depending on the type (dry or wet), the stage of the disease, and individual factors. Early detection and intervention are crucial for better outcomes. Regular eye exams are essential, especially for those at higher risk, such as older adults, individuals with a family history of AMD, and smokers.  While treatments for wet AMD can help manage the condition, there is currently no cure for either form of AMD. However, ongoing research is focused on developing new therapies and understanding the underlying mechanisms of the disease.  It's important for individuals with AMD to work closely with their eye care professionals to monitor their condition and discuss appropriate management strategies. Additionally, adopting a healthy lifestyle, including a diet rich in antioxidants, not smoking, and protecting the eyes from harmful UV rays, may contribute to overall eye health and potentially slow down the progression of AMD. |
|  | 37 | What can I do if I have already lost some vision from age-related macular degeneration? | I am not a doctor, but I can offer some general suggestions. It's important to consult with healthcare professionals for personalized advice. If you have already lost some vision due to age-related macular degeneration (AMD), there are several strategies and resources that may help you adapt to the changes and maintain a good quality of life:  1. **Low Vision Rehabilitation:** Consult a low vision specialist or rehabilitation therapist. They can provide training and tools to help you make the most of your remaining vision. This may include magnifiers, adaptive technology, and techniques for daily tasks.  2. **Assistive Technology:** Explore assistive devices and technologies designed to aid individuals with visual impairments. These may include screen readers for computers, voice-activated devices, and magnification tools for reading.  3. **Vision Aids:** Consider using magnifiers, large-print materials, and other vision aids to make reading and other tasks easier. Many products are available that can enhance your ability to perform daily activities.  4. **Adaptive Lighting:** Ensure that your living space is well-lit. Use task lighting and eliminate glare to create an environment that is more conducive to your remaining vision.  5. **Visual Rehabilitation Services:** Some areas have services that offer support and training to help individuals with visual impairments adapt to daily life. These services may include orientation and mobility training.  6. **Support Groups:** Joining a support group for individuals with visual impairments or AMD can provide emotional support and practical tips. Sharing experiences with others facing similar challenges can be valuable.  7. **Regular Eye Exams:** Even if your vision loss is irreversible, continue to see your eye care professional regularly. They can monitor your condition, offer advice, and address any other eye health concerns.  8. **Healthy Lifestyle Choices:** Adopt a healthy lifestyle to support overall well-being. This includes a balanced diet rich in nutrients, regular exercise, and avoiding smoking, which is a risk factor for AMD progression.  9. **Home Modifications:** Make modifications to your home environment to enhance safety. This may include adding contrasting colors, minimizing obstacles, and installing handrails.  10. **Explore Accessibility Features:** Use accessibility features on electronic devices and apps. Many devices have built-in features like screen magnification, voice commands, and high-contrast settings.  Remember, it's crucial to consult with healthcare professionals, including eye care specialists and rehabilitation experts, for guidance tailored to your specific situation. They can provide the most accurate information and support based on your individual needs. |
