# Supplementary material for: ChatGPT-4 for addressing patient-centred frequently asked questions in age-related macular degeneration clinical practice
Source: Eye (Lond). 2025 Apr 15;39(10):2023–30. doi: 10.1038/s41433-025-03788-0 (PMC12209409; doi:10.1038/s41433-025-03788-0)
Supplement: Supplementary file 8 — Supplementary Table 2 [file 41433_2025_3788_MOESM8_ESM.docx]

| Supplementary Table 2: Free-text comments from evaluators (where written) in response to questions where the average Likert score was less than 4 across three or more quality domains. Comments were taken verbatim unless otherwise stated, with only minor grammatical corrections for readability in this Table. | |
| --- | --- |
| Question 13 | - Needs to remove genetic counselling as it is not currently useful - I would suggest removing the statement regarding genetic counselling unless there is a clear Mendelian cause for the ARMD which is not the case. Given that patients are older it does not have impact on family planning and there is no effective treatment except screening - I am not sure genetic counselling section is useful and UV light exposure is not fully correct. - Rarely AMD is due to genetics and unless there are unusual features I would not refer for genetic testing routinely and this information could create unnecessary anxiety - Genetic counselling in AMD seems unnecessary |
| Question 18 | - Many of these tests are old and not routinely used (e.g. ICG); no mention of OCT-A; genetic testing is not useful; "drusen exam" and "RPE analysis" seem to be taken from proprietary OCT analyses and are not an accepted clinical term - Drusen exam and RPE analysis made to sound separate to eye examination (patients may have the erroneous idea that this can be requested separately routinely) - Don't know what a drusen exam or RPE analysis is - ICG rarely used in AMD - Genetic tests not recommended as AMD multi-genetic, unless IRD suspected (e.g. Sorsby), which is not AMD” - Wouldn’t advocate for genetic testing to the public - Not sure what is meant in 5. other tests - all of those are already covered in the OCT and clinical exams" - Value of genetic testing is limited - wouldn't recommend it for patients that just have family history. - Overall okay answer but should probably mention that FA/ ICG/ genetic testing is not routinely done to diagnose AMD especially in early stages. Probably should be worded that additional testing such as FA, ICGA may be performed in latter stages of the condition. Otherwise could cause some patient anxiety. |
| Question 19 | - Guidelines are probably necessarily vague and broad because of the diversity of possible clinical presentations - The regular eye examination schedule does not seem to be based on Australian Medicare schedules, which suggest 3 yearly for <65 years of age and yearly for 65 years or older. In general optometric practice, eye tests are generally recommended annually to every 2 years for individuals <65 years as well. For AMD, the review schedule could also be shorter than every 6 months if at very high risk of conversion or with current treatment. - Follow up could be as frequent as 4 weekly in wet AMD since they will be returning for injections - Example given for time frame of review for patients with AMD primarily applies to those with dry AMD rather than wet AMD where they need shorter review periods. - Very prescriptive probably best to have more relaxed timeframes for reviews - The guidelines for eye test frequency seems a bit long - e.g. up to 4 years for a 40-54 year-old |
| Question 21 | - Poor because it does not frame the statements with specific criteria, such as driving standards (purposefully vague and broad); for example, it does not necessarily capture people with early AMD who may be fine for driving; it also does not capture issues related to low contrast situations, such as night time driving, and adding restrictions to licencing - I think it is important to mention that it may not be safe to drive, and indeed you may not meet driving standards for vision, in which case this needs to be reported to the driving authority - Might be good to say that a good proportion of people w AMD can still drive - This one is poor. Stating vision standards with examples would be useful. Second, in many jurisdictions, driving with bionic telescopes is possible providing a means to meet driving standards. - If low vision aids or vehicle adjustments were required this person would not be fit to drive. |
| Question 24 | - AREDS 2 needs to be mentioned, especially in the context of smokers and risk of lung cancer with the original formulation; also, laser is a highly targeted therapy and may be dangerous in many patients depending on their phenotype of AMD - I think it would be worth mentioning AREDS2 as patients may look up AREDS and may not be aware of increased risk of beta-carotene with lung cancer for smokers - Not up to date with pegcetacoplan and avacinacaptad pegol being used to treat GA - Neovascularisation [as stated] means it's not dry anymore.... so anti VEGF and laser are not treatments - Anti-VEGF [incorrectly] mentioned can be used to treat dry AMD - should be complement inhibitors rather than anti-VEGF - Anti-VEGF is not indicated for dry AMD however the output suggests that for more advanced cases of dry AMD it is an option. Additionally, it suggests that wet AMD is a complication of dry AMD. - Suggest - monitor for evidence of other eye disease such as cataracts and glaucoma which may worsen peripheral vision with time - Overall okay but slightly inconsistent in its details, e.g. laser for advanced dry AMD. could mention new treatments for late (atrophic) AMD? |
| Question 26 | - This lumps a lot of treatments together and should really clearly state that some of these interventions (temporarily) halt progression, whilst others may partially recover some vision (such as anti-VEGF); "certain surgical procedures" seems vague and not commonly used - Laser is harmful - Surgical procedure for AMD is harmful (not withstanding a very minority of cases of subretinal haem) - still probably not too beneficial" - There is no mention of partial improvement in vision with treatments of AMD, which would have been useful to include in 'treatments available to manage the condition' - Suggest removal surgical procedures - may confuse patients |
| Question 27 | - This may be a product of the older information database, but there needs to be some acknowledgement of emerging treatments that may be injectable for the management of atrophic AMD - Complement inhibitor intravitreal injections available for GA - There are now injections available for geographic atrophy so can sometimes get injection in dry type too - Lacks information on AMD stages |
| Question 28 | - Same comment as previous regarding laser: “laser is a highly targeted therapy and may be dangerous in many patients depending on their phenotype of AMD” - Laser is a harmful treatment for nAMD - A general trend so far is that the chatbot output tends to steer away from definites (e.g. it will say "not all cases of X require Y" when in actuality, NONE of X require Y). - Covers common questions with laser for retinal problems |
| Question 31 | - Miniature telescopes seem to be rarely used and this should be reflected somewhere (such as a stepwise approach); gene therapies could also be mentioned as emerging treatments - Miniature telescopes are used infrequently - should be mentioned. No mention of gene therapy - would make this more comprehensive - Suggest remove the implantable telescope - not mainstay practise and still very experimental - Never heard of this implantable telescope. |
